# Sparse short-distance connections enhance calcium wave propagation in a 3D model of astrocyte networks

**DOI:** 10.3389/fncom.2014.00045

**Published:** 2014-04-16

**Authors:** Jules Lallouette, Maurizio De Pittà, Eshel Ben-Jacob, Hugues Berry

**Affiliations:** ^1^EPI Beagle, INRIA Rhône-AlpesVilleurbanne, France; ^2^LIRIS, UMR 5205 CNRS-INSA, Université de LyonVilleurbanne, France; ^3^School of Physics and Astronomy, Tel Aviv UniversityRamat Aviv, Israel; ^4^Center for Theoretical Biological Physics, Rice UniversityHouston, TX, USA

**Keywords:** glial cells, astrocytes, gap-junctions, wave propagation, network topology

## Abstract

Traditionally, astrocytes have been considered to couple via gap-junctions into a syncytium with only rudimentary spatial organization. However, this view is challenged by growing experimental evidence that astrocytes organize as a proper gap-junction mediated network with more complex region-dependent properties. On the other hand, the propagation range of intercellular calcium waves (ICW) within astrocyte populations is as well highly variable, depending on the brain region considered. This suggests that the variability of the topology of gap-junction couplings could play a role in the variability of the ICW propagation range. Since this hypothesis is very difficult to investigate with current experimental approaches, we explore it here using a biophysically realistic model of three-dimensional astrocyte networks in which we varied the topology of the astrocyte network, while keeping intracellular properties and spatial cell distribution and density constant. Computer simulations of the model suggest that changing the topology of the network is indeed sufficient to reproduce the distinct ranges of ICW propagation reported experimentally. Unexpectedly, our simulations also predict that sparse connectivity and restriction of gap-junction couplings to short distances should favor propagation while long–distance or dense connectivity should impair it. Altogether, our results provide support to recent experimental findings that point toward a significant functional role of the organization of gap-junction couplings into proper astroglial networks. Dynamic control of this topology by neurons and signaling molecules could thus constitute a new type of regulation of neuron-glia and glia-glia interactions.

## 1. Introduction

Astrocytes, one type of glial cells of the brain, respond to neighboring neuronal activity by increases of their cytoplasmic Ca^2+^ concentration (Zonta and Carmignoto, 2002; Agulhon et al., 2008). Such transient intracellular Ca^2+^ signals are generally accepted to trigger the release from the astrocytes of signaling molecules (i.e., “gliotransmitters” like glutamate or ATP) that may regulate neuronal activity (Haydon and Carmignoto, 2006; Perea et al., 2009). Although the molecular pathways supporting these mechanisms remain partly under debate (Agulhon et al., 2010; Sun et al., 2013), their existence suggests a possible signaling role for astrocytes in brain communication, implying that brain information could travel not just in the neuronal circuitry but in an expanded neuron–astrocyte network (Haydon, 2001; Nedergaard et al., 2003; Volterra and Meldolesi, 2005; Giaume et al., 2010).

Calcium elevations can propagate within astrocyte populations as intercellular Ca^2+^ waves (ICWs) (Haydon, 2001; Scemes and Giaume, 2006; Zorec et al., 2012) and these ICWs have extensively been observed in astrocyte cultures. Recent experiments on brain slices as well as *in vivo* (Zorec et al., 2012), confirmed their existence in physiological conditions (Kuga et al., 2011). Much effort has been devoted to understand the biochemical mechanisms responsible for initiation and propagation of ICWs (Charles, 1998; Scemes et al., 2000; Charles and Giaume, 2002). Within the single astrocyte, intracellular Ca^2+^ dynamics is mainly due to Ca^2+^-induced Ca^2+^ release (CICR) from the endoplasmic reticulum (ER) stores—a self-amplifying release mechanism triggered and regulated by inositol 1,4,5-triphosphate (IP_3_) (Nimmerjahn, 2009). On the other hand, although experimental protocols monitor it as variations of intracellular Ca^2+^, the signal that is transmitted from one astrocyte to another in an ICW is generally not Ca^2+^, but ATP or IP_3_ (Scemes and Giaume, 2006). In the first case, the release of ATP from one astrocyte into the extracellular space activates purinergic receptors on neighboring astrocytes, which leads to Ca^2+^ elevations therein (Guthrie et al., 1999; Arcuino et al., 2002). In the second scenario instead, Ca^2+^-increase in the source cell favors IP_3_ production by phospholipase Cδ (PLCδ). Direct IP_3_ transport from the cytoplasm of this cell to the cytoplasm of a coupled astrocyte through gap junction channels (GJCs) then triggers CICR and Ca^2+^ increase in the coupled astrocyte (Venance et al., 1997; Giaume and Venance, 1998; Goldberg et al., 2010). Although the ATP and IP_3_ signaling pathways for ICWs are not mutually exclusive, several lines of evidence suggest that direct IP_3_ diffusion through GJCs is likely the predominant route for propagation in many astrocyte types and brain areas (Carmignoto, 2000; Kettenmann and Ransom, 2004).

The spatial arrangement of astrocytes *in vivo* remains largely unclear. Early reports pointed that astrocytes form non-overlapping domains that “tile” the brain space (Bushong et al., 2002). This suggests a regular spatial arrangement of the cells (Barthélemy, 2010) and leads to a proximity-based coupling rule whereby each astrocyte would be GJC-coupled only to its nearest neighbors, at the boundary of their respective non-overlapping domains. However, more recent data suggested more complex coupling rules (Schipke et al., 2008; Giaume et al., 2010; Roux et al., 2011). Local variability of the coupling organization was reported in the olfactory glomeruli (Roux et al., 2011) or the somatosensory cortex (Houades et al., 2008). More generally, a significant number of the astrocytes found within a given coupling domain are not GJC-coupled to the main astrocyte coupling network. (Houades et al., 2006, 2008; Rela et al., 2010). This indicates that the rule deciding whether two astrocytes are GJC coupled is not purely based on their distance but may be more finely organized into precise anatomical and functional compartments (Pannasch and Rouach, 2013).

One possible effect of the heterogeneity of GJC couplings organization could be a variability in the propagation range of ICW. Indeed, experimental reports of the number of astrocytes activated by a single ICW yield highly variable numbers, from a few cells (Sul et al., 2004; Sasaki et al., 2011) up to 30 (Tian et al., 2006) or even hundreds of cells (Kuga et al., 2011). These discrepancies persist even when the variability due to the type of stimulation employed is factored out (Scemes and Giaume, 2006). This leads to the hypothesis that the variability of the ICW propagation range could be explained by variations of the organization of GJC couplings. The experimental investigation of this hypothesis is however severely limited by the difficulty to distinguish experimentally between variations in the intracellular signaling parameters (enzyme activities, receptor densities…) and variations of the spatial organization of GJC couplings. In such a situation, computer simulations are highly useful since one can easily vary intracellular signaling parameters while guarantying constant GJC-couplings and vice-versa.

Accordingly, computer simulations have been employed in previous studies to investigate how the ICW propagation range depends on the astrocyte-to-astrocyte variability of intracellular signaling parameters, including local IP_3_ regeneration, receptor subtypes, affinity of IP_3_ receptor-channels on Ca^2+^ stores or kinetics of IP_3_ transport through gap-junctions (Höfer et al., 2002; Iacobas et al., 2006; Goldberg et al., 2010). However, in these studies, the astrocytes are generally positioned in a one or two-dimensional space. The organization of GJC couplings in these simulation studies is usually unique and consists in nearest-neighbor coupling (or a variant thereof), in agreement with the hypothesis of non-overlapping domains. In light of the growing evidence that GJC coupling in astrocyte networks may be more complex, the hypothesis that the variability of the ICW propagation range may be due to variations of the organization of GJC couplings, needs to be tested in three-dimensional astrocyte networks with a variety of complex GJC-coupling organizations.

Here, we used computer simulations of ICW propagation in three-dimensional networks of GJC-coupled astrocytes using a realistic biophysical model for astrocytic Ca^2+^ dynamics (De Pittà et al., 2009; Goldberg et al., 2010). Our simulations suggest that changing the organization of GJC couplings is enough to reproduce the large variability of the range of ICW propagation observed in experiments. Therefore, our simulations predict that zones with distinct GJC-coupling should be expected to support ICW with distinct propagation ranges. Moreover, they hint that ICW propagation is favored in astrocyte networks that are sparsely GJC-coupled and that display large mean shortest paths, as found when the GJC couplings are restricted to short Euclidean distances. These results suggest that the principles whereby signals travel in astrocyte networks are different from those at play in neuronal networks.

## 2. Methods

### Astrocyte network model

Intracellular calcium dynamics in the cytoplasm of astrocytes can be described by the *ChI* model that we previously developed and studied. This model provides a realistic description of the dynamics in isolated astrocytes (De Pittà et al., 2009; Goldberg et al., 2010). In this model, possible spatial non-homogeneities of the intracellular distribution of chemical species are neglected. Similarly, the intricate and complex shape of the astrocytes is not taken into account. Astrocytes are thus simplified in the model as perfectly-stirred cells with spherical shapes. Albeit a crude approximation, this allows simulating ICW propagations in large astrocyte populations (>10^3^ cells), an objective that is much harder to achieve with more detailed description of intracellular dynamics. The *ChI* model considers both Ca^2+^ regulation by IP_3_-dependent CICR as well as IP_3_ dynamics resulting from PLCδ–mediated production and degradation both by IP_3_ 3-kinase (3K) and inositol polyphosphate 5-phosphatase (5P). Accordingly, the intracellular Ca^2+^ dynamics in the *i*-th astrocyte of the network is described by three coupled non–linear ordinary differential equations, for each astrocyte number *i* = {1, …, *N*}:

(1) $$\frac{\text{d}}{\text{d}t}C_{i}=J_{C}(C_{i},h_{i},I_{i})+J_{L}(C_{i})-J_{P}(C_{i})$$

(2) $$\frac{\text{d}}{\text{d}t}h_{i}=\Omega_{h}(C_{i},I_{i})(h_{\infty }(C_{i},I_{i})-h_{i})$$

(3) $$\frac{\text{d}}{\text{d}t}I_{i}=J_{\delta }(C_{i},I_{i})-J_{3K}(C_{i},I_{i})-J_{5P}(I_{i})+J_{i}^{diff}$$

where the variables *C*_*i*_,_,_*h*_*i*_, *I*_*i*_ respectively denote the cell-averaged cytosolic Ca^2+^ concentration, the fraction of activable IP_3_R channels on the ER membrane, and the cell-averaged cytosolic IP_3_ concentration. In this model, calcium dynamics (Equation 1) are the results of the interplay between three fluxes: a Ca^2+^ uptake from the cytosol to the ER (*J*_*P*_); a passive Ca^2+^ leak from the ER to the cytosol (*J*_*L*_); and an IP_3_-mediated Ca^2+^ efflux from the ER to the cytosol (*J*_*C*_). The fraction of activable IP_3_R channels (Equation 2) relaxes with a Ca^2+^ and IP_3_ dependent rate (Ω_*h*_) to an equilibrium value *h*_∞_. Finally, the IP_3_ concentration dynamics (Equation 3) are defined by the balance between IP_3_ production by PLCδ (*J*_δ_) and degradation by 3K (*J*_3*K*_) and 5P (*J*_5*P*_). The additional term *J*^*diff*^_*i*_ sums IP_3_ flows (*J*_*ij*_) from/to any cell *j* that is directly connected (GJC-coupled) to cell *i*, i.e., *J*^*diff*^_*i*_ = ∑_*j*∈
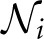
*i*_ *J*_*ij*_ with 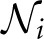
_*i*_ the set of astrocytes that are GJC-coupled to *i* (Goldberg et al., 2010). In terms of cell-averaged concentrations, the transport of IP_3_ between two cells may be more complex than simple linear diffusion through GJCs (Nagy and Rash, 2000). It could actually be regarded as a multiscale phenomenon that depends on many factors, including cell morphology and GJC location, permeability and physiology (De Pittà et al., 2012). To account for these factors, we assumed non–linear IP_3_ transport between GJC-coupled astrocytes, as detailed in the Supplementary Material S1, with

(4) $$J_{ij}=-\frac{F}{2}\left(1+\tanh\left(\frac{|\Delta_{ij}I|-I_{\theta }}{\omega_{I}}\right)\right)\frac{\Delta_{ij}I}{\left|\Delta_{ij}I\right|}$$

where Δ_*ij*_*I* = *I*_*i*_ − *I*_*j*_ represents the IP_3_ gradient between cells *i* and *j, I*_θ_ represents the threshold IP_3_ gradient for effective intercellular diffusion, i.e., the minimal IP_3_ gradient allowing effective IP_3_ transfer. ω_*I*_ sets the slope of the increase of the flux with Δ_*ij*_*I*. Finally, the parameter *F* corresponds to the maximal diffusion flux between cells *i* and *j*, and may thus be regarded as the strength of coupling between two cells. Note however that our results do not crucially depend on this non–linear coupling, since linear (diffusive) couplings yields qualitatively similar results (see Supplementary Figure S7). A detailed presentation of the model can be found in the Supplementary Material S1.

### Topologies of the GJC-coupled astrocyte networks

Just like neurons, astrocyte-astrocyte signaling interactions via GJC coupling can be modeled as a network, where each node represents an astrocyte (whole cell) and the links locate GJC–mediated connections (couplings) between two astrocytes (Giaume et al., 2010). To address ICW propagation in realistic conditions, we constructed three-dimensional spatial networks of astrocytes by a two-step procedure. First *N* astrocytes were properly positioned in the physical (Euclidean) space to match experimental data on astrocyte spatial arrangement. Then, a wiring strategy was deployed to establish coupling among the astrocytes to obtain networks of desired coupling organizations.

**Figure 1:**
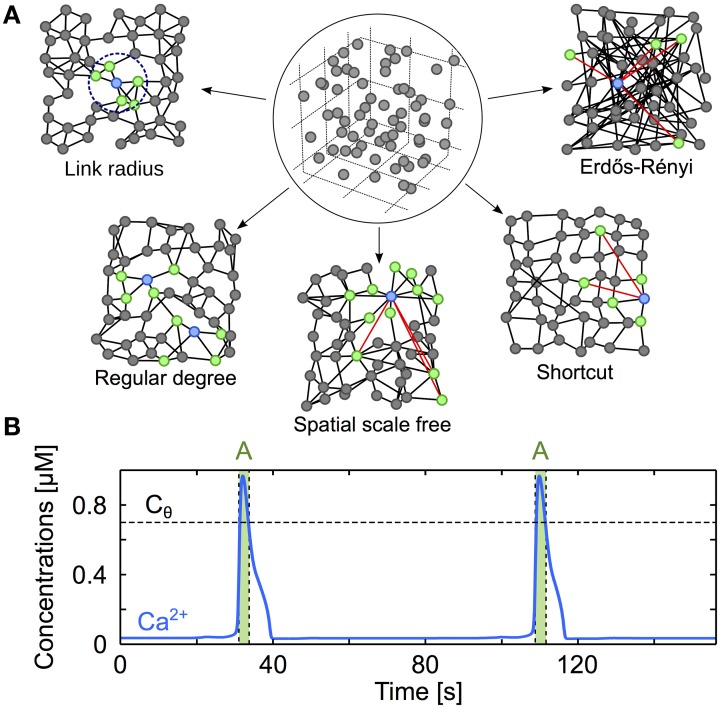
**Modeling three-dimensional astrocyte networks. (A)** The astrocytes (*circles*) are first positioned on a cubic lattice. The positions are then jittered by random values so as to reproduce experimentally-derived cell distance statistics (*circled panel*). The astrocytes are then GJC-coupled (*black lines*) according to different coupling rules (described in “Methods”), yielding distinct types of coupling organizations (*other panels*). For clarity, the networks illustrated here are two-dimensional, but the networks used in the study are systematically three-dimensional. *Red links* denote shortcuts, *green* cells locate the cells that are GJC-coupled to the *blue* one. **(B)** Typical trace from simulation of the *ChI* model that gives the intracellular Ca^2+^ dynamics in each cell of the network. Astrocytes are considered activated (A, *green-shaded regions*) when their cytosolic Ca^2+^ concentration (*blue traces*) exceeds the threshold *C*_θ_ = 0.7 μM (*black dashed lines*). Astrocyte parameters as in Table 1.

To achieve proper positioning of astrocytes in space, *N* = 11^3^ model astrocytes were initially positioned on a 11 × 11 × 11 cubic lattice with internode distance *a* (Figure 1A, *circled panel*). The position of each astrocyte was then jittered by Gaussian noise with zero mean and variance σ^2^ to reproduce the experimental measurements of the distribution of astrocyte-to-astrocyte distances in the mouse hippocampus: the distance between nearest–neighbor cell centers was reported to be 50 μm on average with a minimal value of 20 μm and a coefficient of variation ~0.25 (Sasaki et al., 2011). Using minimization of square errors, we could reproduce these experimental data in our model using a cubic lattice internode distance *a* ≈ 70 μm followed by a Gaussian jitter with σ^2^ = 55^2^ μm^2^. Therefore, the simulation comprised *N* = 1331 astrocytes in a domain of size ≈ 0.50 mm^3^.

With astrocytes properly positioned, we implemented different organizations of the network of GJC couplings. Because the organization of GJC couplings *in vivo* remains uncertain, several plausible coupling networks were considered (see main text and Figure 1A):

1. *Regular degree* networks were constructed by coupling each astrocyte to its *k* nearest neighbors in space, where *k* is a parameter that we systematically varied. Each astrocyte in these networks is thus GJC-coupled to exactly the same number of cells (*k*).
2. *Link radius* networks are a subtle variant: they are constructed by coupling each astrocyte to all the cells that are found within a distance *d*, where *d* is a parameter that we varied here. Therefore, the number of distinct cells coupled to each astrocyte in link radius networks is a random variable, whose average value (〈*k*〉) increases with *d*.
3. *Shortcut* networks are cubic lattices in which a low amount of long distance GJC coupling is allowed. We started from a cubic lattice that we built with an internode distance *a*. We then linked each node to his nearest neighbors at distances that are multiples of *a* and lower or equal to *a* × *m*_latt_. As we work in a *d*′ = 3 three-dimensional space, the mean degree of a node can only take discrete 2*m*_latt_*d*′ values. Detailed description of these lattices can be found in Watts (1999). Node position were then jittered as explained before and each GJC coupling was rewired with probability *p*_*s*_ (a parameter that was varied): that is, with probability *p*_*s*_, one of the edge ends of each GJC coupling was replaced by a (uniformly) randomly chosen cell. These networks bear so–called “small–world” features depending on the value of *p*_*s*_ (Albert and Barabási, 2002).
4. *Spatial scale free* networks were introduced to test the influence of “hub” astrocytes, i.e., astrocytes coupled to a very large number of other cells (over 20 neighbors, that is twice as much as the estimated mean degree in 3D astrocyte networks *in situ* Xu et al., 2010). These networks were incrementally built by a classical preferential attachment rule, where one introduces astrocytes one after the other in the network and preferentially connects them to the astrocytes with larger degree yet considering spatial distances, according to the procedure described in Barthélemy (2010). More precisely, each newcomer astrocyte is coupled to *m*_*sf*_ astrocytes of the network formed by the previously coupled astrocytes. These *m*_*sf*_ astrocytes are randomly chosen but with the constraint that the probability *p*_*i→j*_ to choose astrocyte *j* increases with *j*′s degree and decreases with its distance: *p*_*i→j*_ ∝ *k*_*j*_ exp(−*d*_*ij*_/*r*_*c*_) where *i* is the index of the newcomer and *d*_*ij*_ the distance between *i* and *j*. The parameter *r*_*c*_ controls the trade-off between scale–free structure and the restriction of the couplings to short distances (Barthélemy, 2010). For large values of *r*_*c*_ (larger than 20 μm in our case), some of the astrocytes (“hubs”) are coupled to a very large number of cells but the GJC couplings extend over large distances. On the contrary, small *r*_*c*_ values (less than 5 μm in our case) lead to networks that are devoid of hubs but that feature short distance GJC couplings.
5. *Erdős-Rényi* networks were constructed by coupling each pair of astrocytes with probability *p*, independently of their distance. Hence, in these networks the distance between astrocytes is not a constraint for their GJC coupling. They have mostly been used here as a control to test our theories about what quantifiers of the network organization conditions ICW propagation.

While both regular degree and link radius networks may be regarded as spatially–constrained networks inasmuch as the coupling between the astrocytes are limited by the distance between cells, the presence of long-distance GJC couplings in shortcut and Erdős-Rényi networks, makes these networks essentially spatially–unconstrained.

### Numerical methods

Simulations of Ca^2+^ propagation in astrocyte networks were performed by numerical integration of the network model by a fourth order Runge–Kutta scheme with a time step of 0.01 s. The parameters of the *ChI* model Equations (1–3), (reported in Table 1) were chosen according to previous studies (De Pittà et al., 2009; Goldberg et al., 2010) so as to reproduce the experimentally-observed pulse-like shape (Figure 1B) of propagating ICW waveforms (namely Ca^2+^ pulses of width much smaller than their wavelength) and whose frequency increases with the frequency or the intensity of stimulation (Pasti et al., 1997; Tian et al., 2006; Kuga et al., 2011). The organization parameters (e.g., *k, d, r*_*c*_, *p*_*s*_, *p*) were systematically varied so as to obtain networks with a mean degree 〈*k*〉 ranging from 3 to 17 (reported in Table 2). The minimal value of 〈*k*〉 was set by the constraint that the fraction of node pairs with infinite topological distance should be below 2%. The coupling organizations used here are essentially random networks so that each organization parameter defines a *distribution* of coupling networks. For statistical significance, quantification of ICW propagation in these coupling networks must therefore be averaged over several samples of the random networks defined by a given coupling organization type and a given parameter. Here, simulation results were averaged over 20 different network samples for each value of the organization parameter and each organization class. Ca^2+^ wave propagation was triggered by selectively stimulating an astrocyte of the network for all the duration *T* = 200 s of the simulation. This allowed Ca^2+^ waves to fully propagate to their maximum extent (see Supplementary Material S1). During simulations, an astrocyte was considered activated if its cytosolic Ca^2+^ concentration exceeded a threshold value of *C*_θ_ = 0.7 μM (Figure 1B). As calcium oscillations are very stereotypical with the parameters that we chose, significant calcium oscillations always crossed this threshold. The extent of Ca^2+^ ICW propagation was quantified by the total number of cells (*N*_act_) that were activated at least once during a simulation. To minimize boundary effects due to the spatial confinement of the modeled network, stimulation was delivered to an astrocyte located in the center of the network.

**Table 1:** **Biochemical parameters of the astrocyte network model**.

**Symbol**	**Description**	**Value**	**Units**
**IP_3_R KINETICS**			
*d*_1_	IP_3_ binding affinity	0.13	μM
*O*_2_	Inactivating Ca^2+^ binding rate	0.2	μM^−1^s^−1^
*d*_2_	Inactivating Ca^2+^ binding affinity	1.049	μM
*d*_3_	IP_3_ binding affinity (with Ca^2+^ inactivation)	0.9434	μM
*d*_5_	Activating Ca^2+^ binding affinity	0.08234	μM
**CALCIUM FLUXES**			
*C*_*T*_	Total ER Ca^2+^ content	2	μM
ρ_*A*_	ER-to-cytoplasm volume ratio	0.185	–
Ω_*C*_	Maximal Ca^2+^ release rate by IP_3_Rs	6	s^−1^
Ω_*L*_	Maximal Ca^2+^ leak rate	0.11	s^−1^
*O*_*P*_	Maximal Ca^2+^ uptake rate	0.9	μM s^−1^
*K*_*P*_	Ca^2+^ affinity of SERCA pumps	0.05	μM
**IP_3_ PRODUCTION**			
*O*_δ_	Maximal rate of IP_3_ production by PLCδ	0.7	μM s^−1^
*K*_δ_	Ca^2+^ affinity of PLCδ	0.1	μM
κ_δ_	Inhibiting IP_3_ affinity of PLCδ	1.5	μM
**IP_3_ DEGRADATION**			
Ω_5*P*_	Maximal rate of IP_3_ degradation by IP-5P	0.21	s^−1^
*O*_3*K*_	Maximal rate of IP_3_ degradation by IP_3_−3K	4.5	μM s^−1^
*K*_*D*_	Ca^2+^ affinity of IP_3_-3K	1	μM
*K*_3*K*_	IP_3_ affinity of IP_3_-3K	0.7	μM
**IP_3_ DIFFUSION**			
*F*	GJC IP_3_ permeability (linear)	2	s^−1^
	GJC IP_3_ permeability (non–linear)	2	μM s^−1^
*I*_θ_	Threshold IP_3_ gradient for diffusion	0.3	μM
ω_*I*_	Scaling factor of diffusion	0.05	μM
*I*_*bias*_	IP_3_ bias	2	μM
**SIMULATION**			
*T*	Simulation time	200	s
*t*_*s*_	Stimulation time	200	s

*The full model equations are given in Supplementary Material S1*.

**Table 2:** **Spatial and topological parameters of the astrocyte network model**.

**Symbol**	**Description**	**Min**	**Values**	**Max**	**Units**
			**step**		
**SPATIAL ORGANIZATION**					
*a*	Internode distance	70			μm
σ	Variance of the gaussian noise	55			μm
**NETWORK TOPOLOGY**					
*k*	Degree of regular networks	3	1	15	-
*d*	Linking distance for link radius networks	80	5	120	μm
*r*_*c*_	Spatial control parameter for spatial scale free networks	2	1	4	μm
-	-	5	20	105	μm
*m*_*sf*_	Number of new links for spatial scale free networks	2	1	5	-
*m*_latt_	Internode linking distance factor for shortcut networks	1	1	3	-
*p*_*s*_	Probability of rewiring an edge for shortcut networks	0	0.02	0.1	-
-	-	0.2	0.1	0.4	-
*p*	Linking probability for Erdős-Rényi networks	$\frac{5}{N-1}$	$\frac{1}{N-1}$	$\frac{15}{N-1}$	-

### Theoretical analysis

In order to identify the major properties conditioning the ICW propagation range, we have carried out mathematical analysis of the computational model, focusing on the effect of network structure. This analysis is detailed in Supplementary Material S2.

## 3. Results

### The spatial organization of GJC couplings drives the extent of ICW propagation

To investigate the effect of network organization on ICW propagation, we developed a computer model that simulates ICWs propagation in three-dimensional astrocyte networks. In the model, each astrocyte is a point-like object described by its location in space and three internal variables (see Methods): its cytoplasmic Ca^2+^ concentration *C*, the fraction *h* of activable IP_3_R channels in the membranes of Ca^2+^ stores and its cytoplasmic IP_3_ concentration *I*. Therefore we neglect complications due to the intricate shape of astrocytes. The model expresses the evolution in time of each of these quantities taking into account IP_3_-mediated exchanges between internal Ca^2+^ stores and Ca^2+^-dependent IP_3_ synthesis and degradation. To this aim, we used the *ChI* model that provides a realistic description of Ca^2+^-IP_3_ dynamics in isolated astrocytes (De Pittà et al., 2009; Goldberg et al., 2010) (see Methods and Supplementary Material S1 for a detailed description).

In the model, we position *N* = 1331 astrocytes in a three-dimensional domain of size ≈ 800 × 800 × 800 μm^3^ (roughly 0.5 mm^3^) so as to emulate the distribution of cell-cell distances reported in mouse hippocampus (Sasaki et al., 2011) (see Methods). Each astrocyte can be coupled via gap-junction channels (GJCs) to other astrocytes. Such a coupling allows bidirectional transport of IP_3_ directly from cytoplasm to cytoplasm of the two coupled cells. How these gap junction couplings are organized, i.e., what is the rule deciding whether two astrocytes are GJC-coupled in the network (Figure 1A) is referred to as the “topology” of the network and is the main focus of our study. Since the exact organization of couplings in astrocyte networks *in vivo* is still unclear, we have implemented several possible coupling topologies (see Methods). The topologies included (*i*) spatially–constrained link radius or regular degree networks, where astrocyte are GJC-coupled only when they are close in space, and (*ii*) spatially–unconstrained networks, such as Erdős-Rényi networks, where astrocytes are GJC-coupled independently of their distance. In between these two network classes, spatial scale free and shortcut networks feature a parameter (*r*_*c*_ and *p*_*s*_, respectively) that allows to continuously vary them from spatially–constrained to spatially–unconstrained.

To quantify these coupling topologies, we introduce two classical quantifiers of such complex networks (Albert and Barabási, 2002; Barthélemy, 2010): the average number of distinct cells coupled to each astrocyte, 〈*k*〉 (or *mean degree*), that quantifies the well-connectedness of the network, and the *mean shortest path L*. The shortest path (or topological distance) between two astrocytes is the minimal number of GJC couplings one must cross to connect the two astrocytes. *L* is the average of the shortest paths between all astrocyte pairs and quantifies the mean distance between astrocytes in terms of numbers of couplings. Each type of coupling organization listed above comes with one or two construction parameters (*k, d ,p*_*s*_…). We varied these parameters so as to change the values of the mean degree 〈*k*〉 and the mean shortest path *L* for each type of coupling organization. Note that network topologies can be characterized using other quantifiers of complex networks [like the clustering coefficient, the distribution of the degrees *P*(*k*) or the hierarchical clustering coefficient (Boccaletti et al., 2006; Costa and da Rocha, 2006; Feldt et al., 2010)] but a preliminary investigation by these quantifiers did not account for the propagation extent as well as 〈*k*〉 and *L* did (see Supplementary Material S2).

All the above networks differed only by the topology of the GJC couplings since all other parameters were kept constant, including the kinetics of GJC coupling, the number (*N* = 1331), spatial distribution and density of the astrocytes, the parameters of the dynamics inside each astrocyte as well as the stimulation that triggers the ICW (see Methods). Therefore, differences in ICW propagations across different networks can unambiguously be related to differences in coupling organization. In the simulations, the propagation of the ICW to a given astrocyte is characterized by a strong and transient elevation of cytosolic Ca^2+^ in this astrocyte (Figure 1B). We considered that the ICW reaches an astrocyte, or that, equivalently, an astrocyte is activated by the ICW, if the cytosolic Ca^2+^ concentration in the astrocyte exceeds a threshold value of *C*_θ_ = 0.7 μM. To quantify the propagation range of an ICW, we counted the number of astrocytes that got activated at least once (*N*_act_) during the propagation of the ICW.

**Figure 2:**
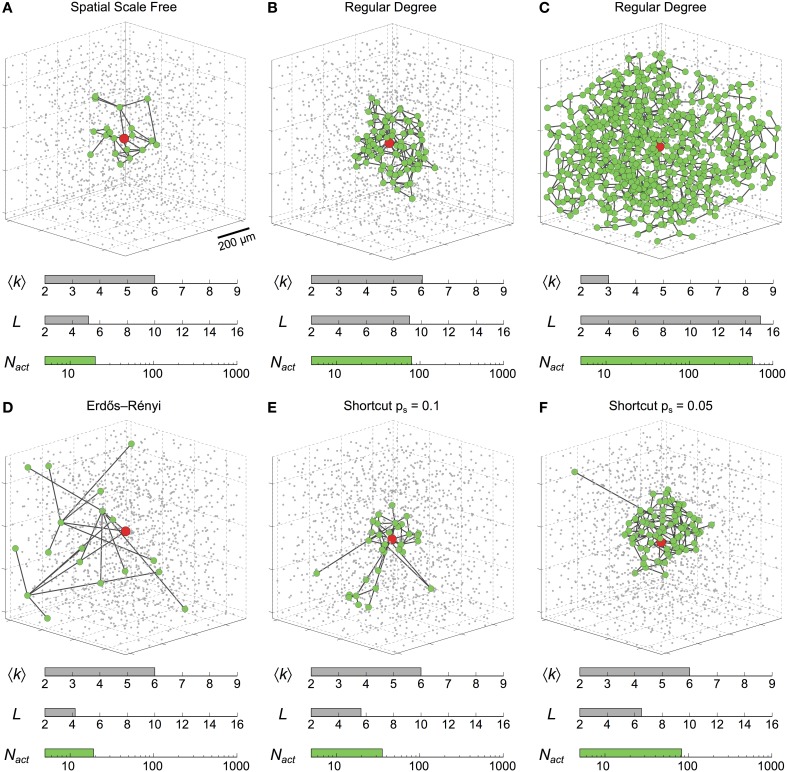
**Intercellular Ca^2+^ waves in 3D astrocyte networks of different coupling organizations**. Changing the organization of the couplings between astrocytes dramatically affects the extent of propagation of ICWs, as quantified by the number of activated astrocytes (*N*_act_). The six networks in this figure feature the same number of cells (*gray dots*). *Green circles* denote astrocytes that were activated by the ICW triggered via stimulation of the *red* astrocyte in the center of the network. **(A)** *r*_*c*_ = 25 μm, *m*_*sf*_ = 3; **(B)** 〈*k*〉 = 6; **(C)** 〈*k*〉 = 3; **(D)** *p* = $\frac{5}{N-1}$; **(E)** *p*_*s*_ = 0.1, *m*_latt_ = 1; **(F)** *p*_*s*_ = 0.05, *m*_latt_ = 1. Astrocyte parameters as in Table 1.

Figure 2 illustrates the type of behaviors observed during typical simulation examples. It shows the cells (*green circles*) that got activated by an ICW triggered by a prolonged stimulation (*t* = 200 s) of the astrocyte shown in *red*. In a spatial scale free network (Figure 2A) with mean degree as large as 〈*k*〉 = 6 and relatively small mean shortest path *L* = 5, ICW propagation was restricted only to *N*_act_ ≈ 20 astrocytes around the stimulated cell. The extent of propagation was considerably larger (i.e., *N*_act_ ≈ 80) in the regular degree network shown in Figure 2B that shares the same mean degree but has larger mean shortest path (i.e., *L* = 9) than the previous one. Remarkably however, in another regular degree network with even larger mean shortest path (*L* = 15) but small mean degree 〈*k*〉 = 3 (Figure 2C), ICW propagated to roughly all the cells in the network, that is *N*_act_ ≈ *N*. Keeping a constant mean degree 〈*k*〉 = 6, increases in mean-shortest path are linked to increases in ICW extent. Indeed, from a purely random Erdős-Rényi network with very low mean-shortest path (Figure 2D) to shortcut networks with 5% rewiring probability (Figure 2F), lowering the amount of shortcuts in a network increases ICW extents. Figures 2A–C are reminiscent of the different types of ICWs observed in experiments: namely, localized Ca^2+^ waves, for which *N*_act_ ≈ 10−50 (Charles, 1998; Sul et al., 2004; Sasaki et al., 2011), as well as long-range propagating ICWs involving a large number of cells (*N*_act_ > 100) (Hirase et al., 2004; Peters et al., 2005; Kuga et al., 2011).

Taken together, the simulations in Figure 2 suggest that the organization of coupling in an astrocyte network can dramatically control the extent of propagation of ICWs. Accordingly, the different ranges of ICW propagation observed in experiments can be partly explained by differences in the organization of GJC coupling between cells in the network. In particular, as in the case of the regular degree networks of Figures 2B,C, the propagation extent seems to critically depend on network characteristics such as the mean degree 〈*k*〉 and the mean shortest path *L*. We investigate this issue in the next section.

### Small mean degrees and large mean shortest paths favor ICW propagation

**Figure 3:**
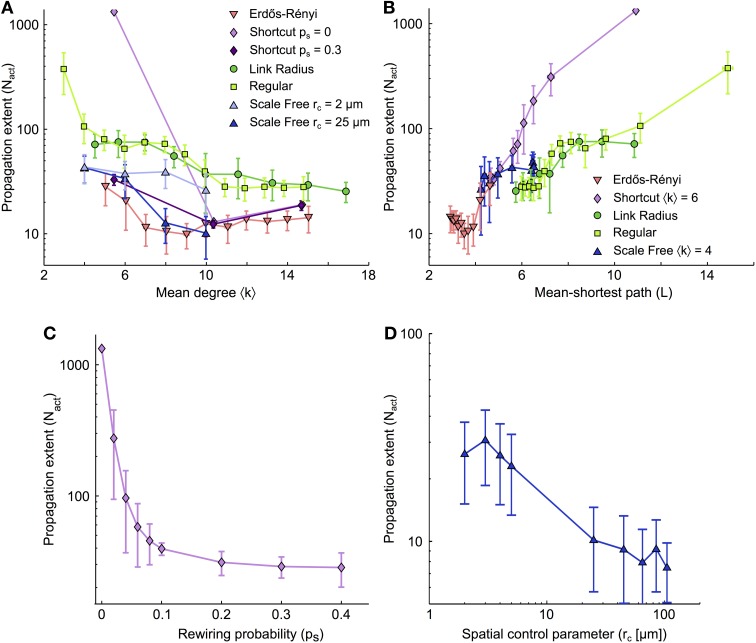
**Dependence of the extent of ICW propagation on the main quantifiers of the coupling organization**. **(A)** Extent of ICW propagation (quantified by the number of activated cells, *N*_act_) as a function of the mean degree 〈*k*〉 and **(B)** of the mean-shortest path *L*. The number of activated cells segregates spatially–constrained (link radius, regular degree and shortcut with *p*_*s*_ = 0) from spatially–unconstrained coupling organizations (shortcut with *p*_*s*_ > 0, and Erdős-Rényi). **(C)** Extent of propagation in shortcut networks as a function of the rewiring probability *p*_*s*_. **(D)** Extent of propagation in spatial scale free networks as a function of the parameter *r*_*c*_ that controls the trade-off between scale–free structure and the restriction of the couplings to short distances. **(C,D)** suggest that ICW propagation is favored by short-distance GJC couplings between astrocytes while long–distance couplings hinder propagation. Simulations as described in Figure 2. Data points ± errorbars correspond to mean values ± standard deviation over 20 sampled networks with the same statistical parameters (see Methods). The shortcut networks in **(C)** were all built with 〈*k*〉 = 6 (i.e., *m*_latt_ = 1). The spatial scale free networks in **(D)** were built with 〈*k*〉 = 10 (i.e., *m*_*sf*_ = 5). Astrocyte parameters as in Table 1.

Figure 3A summarizes the number of cells activated by ICW propagation (*N*_act_) as a function of the mean degree of the network (〈*k*〉) for different types of coupling organization. Although the precise behavior of *N*_act_ vs. 〈*k*〉 depends on the type of organization, the range of ICW propagation follows a generic rule: whatever the type of coupling organization, the propagation range decreases with increasing 〈*k*〉. Therefore, as a general rule, the larger the number of GJC couplings between cells, the worse the propagation. This is at odds with the conclusions from many studies related to dynamics on complex networks, where a large mean degree is usually associated with better signal propagation (Isham et al., 2011). Moreover, for a given value of the mean degree 〈*k*〉, propagation is generally better in networks with only short distance couplings (Link radius, Regular, Shortcut with *p*_*s*_ = 0) than in networks featuring large-distance couplings (Erdős-Rényi, Shortcut with *p*_*s*_ > 0). This can also be seen by comparing scale-free networks with *r*_*c*_ = 2 μm (*light blue*, featuring almost no long-distance coupling) with scale-free networks with *r*_*c*_ = 25 μm (*dark blue*, with many long-distance couplings). The latter essentially supports shorter propagation than the former. On the other hand, for a given 〈*k*〉 value (e.g., 〈*k*〉 = 6), the extent of ICW propagation can differ up to 5–10 folds between coupling organizations. This indicates that the mean degree is not the unique causal quantity, and that other features of the network are likely to regulate ICW propagation.

In Figure 3B, we plot the propagation range *N*_act_ as a function of the mean shortest path *L* for all studied coupling organizations. Here again, a conserved trend is observed: whatever the type of organization, the propagation range *N*_act_ grows with the mean shortest path *L*. And here again, this result contrasts with the common intuition that small mean shortest paths represent large efficiency in signal transmission and should optimize signal propagation in a network (Zanette, 2002). Moreover, from the dependence of *N*_act_ on *L*, one clearly distinguishes the two distinct groups of coupling organizations: organizations that are strictly restricted to short-distance couplings (Link radius and Regular degree) and those that are not (shortcut, Erdős-Rényi). For each group, the curves of *N*_act_ vs *L* essentially collapse on roughly a single curve, so that the increase of *N*_act_ with *L* is described by only two curves for all coupling organizations.

For scale-free networks, we changed the value of the mean shortest path *L* by varying *r*_*c*_: small values of *r*_*c*_ give mostly short-distance couplings, large *L* values and essentially no hubs whereas large values of *r*_*c*_ yield long-distance couplings with small *L* values and highly connected hubs. Figure 3B shows that the propagation range in scale-free networks is mostly given by the presence or absence of long-distance GJC-couplings, independently of the presence of hubs. Indeed, when *L* is small thus long-distance GJC and hubs are present, scale-free networks essentially behave like Erdős-Rényi and Shortcut networks (that display long-distance GJC but no hubs). Therefore the presence of long-distance GJC couplings in scale-free organizations with small *L* seems more influential than the presence of hubs. When *L* increases, scale-free networks, that progressively loose long-range couplings and hubs, crossover to the behavior of Link radius and Regular degree networks, i.e., the coupling organizations that feature only short-distance couplings. This evidences that spatial constraint and mean shortest path are the major topological determinants of ICW propagation, while the presence of hubs, for instance, is less influential. Figure 3C shows the propagation range in shortcut networks for increasing values of rewiring probability *p*_*s*_, i.e., progressively larger numbers of long-distance GJC couplings between astrocytes. The ICW propagation range decays very rapidly with increasing *p*_*s*_ although all networks have the same mean degree. Similarly, in spatial scale free networks, allowing long distance links by increasing *r*_*c*_ leads to decreases in ICW extent (Figure 3D). Hence, long-distance couplings strongly restricts the extent of ICW propagation.

In summary, our analysis suggests that the dependence of ICW propagation in astrocyte networks on mean degree and mean shortest paths is the exact opposite of the common view on signal propagation in complex networks: for all simulated coupling organizations, ICW propagation was improved when either the mean degree was small or the mean shortest path was large and strongly decreased when long-distance couplings were introduced. Note that these organization properties are interrelated, since the strict restriction of GJC coupling to short distances yields large mean shortest paths while the introduction of long-distance GJC coupling reduces the mean shortest path.

### Influence of the shell structure

**Figure 4:**
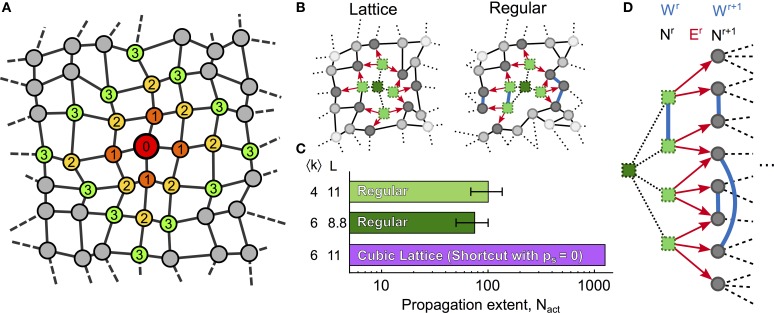
**Shell analysis**. **(A)** Ilustration of shells 1–3 in a generic network; nodes are colored according to their topological distance (*numbers*) from a *red* reference node. A shell is a set of nodes that have the same topological distance *r* to the reference node (thus the same color in the figure). **(B)** 2D representation of ICW propagation (*red arrows*) in a cubic lattice and a regular network with identical mean degree. *Bright green squares* represent activated cells while *dark green squares* denote cells that were previously activated by the ICW. **(C)** Despite sharing similar topological features, propagation in the cubic lattice may activate up to 10-fold more cells than in regular networks. **(D)** Shell analysis reveals that this difference in ICW propagation may be attributable to the connections between cells within the same *r*-th shell (*W*^*r*^) in regular networks, which reduce the quantity of IP_3_ given to astrocytes in the next shell *r* + 1, thus resulting in earlier propagation failures than in cubic lattices. Definitions of *N*^*r*^, *W*^*r*^, and *E*^*r*^ are given in the main text. Cubic lattices were built by shortcut networks with *p*_*s*_ = 0 and *m*_latt_ = 1 (see “Methods”). Model parameters as in Table 1.

A characterization of the features of network topology that locally regulate ICW propagation may be obtained by the analysis of the shell structure of the network (see Figure 4A), where each shell *r* is defined as the set of cells at shortest path (topological) distance *r* from a reference cell that we choose as the cell that initiates the ICW. These shell structures are schematically exemplified in Figure 4B for two network topologies: cubic lattices (〈*k*〉 = 6, *L* = 11), and regular networks either with the same mean degree (〈*k*〉 = 6, *L* = 8.8) or with the same mean shortest path (〈*k*〉 = 4, *L* = 11). The procedures used to generate these networks were very similar: for the cubic lattice, the astrocytes were first connected to their *k* = 6 nearest neighbors, then their positions were jittered (as described in methods), leaving the connections unchanged. Conversely, regular networks were built first by jittering the cell position, and then by connecting the cells to their *k* = 6 nearest neighbors. Those cubic lattice and regular networks share identical topological parameters and the connections between their cells show only subtle differences. Even though these differences are minute, ICW propagation in these networks are remarkably distinct, with a number of cells activated by an ICW (*N*_act_) that is up to ten-fold larger in cubic lattices than in regular networks (Figure 4C). This astonishing behavior can be explained by differences in the shell structure of these networks.

Let us denote by *N*^*r*^ the number of cells in the *r*-th shell, by *W*^*r*^ the number of links between cells within the same *r*-th shell, and by *E*^*r*^ the number of links between cells in shell *r* and cells in next shell *r* + 1. Figure 4D illustrates the three quantities *N*^*r*^, *W*^*r*^, *E*^*r*^ for two consecutive shells. While there are no links between cells belonging to the same shell in cubic lattices, regular networks feature significant amounts of intra-shell connections. Therefore, while *W*^*r*^ = 0 for every shell of the cubic lattice, *W*^*r*^ ≥ 0 in regular networks. This observation crucially accounts for the smaller ICW extent in regular networks compared to cubic lattices.

The forward propagation of an ICW away from its originating cell in fact can be regarded as a shell-by-shell activation process. Astrocytes inside a given shell however do not activate all at the same time so that, when only a fraction ρ of shell *r* is activated (i.e., ρ *N*^*r*^ astrocytes are activated in shell *r*), the IP_3_ quantity that they will produce will be distributed among: (1) *N*^*r*−1^ astrocytes in the preceeding shell *r* − 1; (2) *N*^*r*+1^ astrocytes in the following shell *r* + 1; and (3) ${\hat{N}}^{r}$ unactivated astrocytes linked to the activated ones in shell *r*. This latter quantity can be expressed considering the number of unactivated astrocytes in shell *r* ((1 − ρ)*N*^*r*^) and the probability for an unactivated astrocyte in shell *r* to be connected to an activated astrocyte in the same shell (≈ 2 ρ *W*^*r*^/(*N*^*r*^ − 1), derived in Supplementary Material S3.3). The number of unactivated astrocytes in shell *r* connected to activated ones in the same shell thus reads:

(5) $${\hat{N}}^{r}\approx (1-\rho )N^{r}\times \frac{2\rho W^{r}}{N^{r}-1}\approx 2\rho (1-\rho )W^{r}$$

Assuming that, for each activated astrocyte in shell *r*, the total amount *Q*_0_ of IP_3_ passed along the *k*_*i*_ neighbors is constant (see Supplementary Material S3.1 and Supplementary Figure S6), the total amount of IP_3_ flowing out of the activated astrocytes in shell *r* can be approximated by *Q*_0_ ρ *N*^*r*^. As IP_3_ going out of shell *r* will be divided among the *N*^*r*−1^ astrocytes of the preceding shell, the *N*^*r*+1^ astrocytes of the following shell and the 2ρ(1 − ρ)*W*^*r*^ unactivated astrocytes in the current shell, the mean IP_3_ supply to each of these unactivated astrocytes is then:

(6) $$\Psi_{out}^{r}\approx Q_{0}\frac{\rho N^{r}}{N^{r-1}+N^{r+1}+2\rho (1-\rho )W^{r}}$$

Since *W*^*r*^ = 0 in cubic lattices (see above), equation (6) predicts that the amount of IP_3_ supplied to unactivated astrocytes of shell *r* + 1 should be smaller in regular networks than in cubic lattices. Accordingly, the extent of ICW propagation is predicted to be smaller in regular networks than in the cubic lattices. This prediction agrees well with the simulations summarized in the histogram in Figure 4C (see also Supplementary Materials S3.3).

To conclude, the above analysis shows that the shell structure of the network has a crucial impact on ICW propagation, in addition to the general trends imposed by 〈*k*〉 and *L*. These results can be interpreted so as to define simple propagation rules that summarize the main properties of ICW propagation in astrocyte networks. These rules are given and tested in the last section.

### The range of ICW propagation is dictated by the local balance between IP_3_ accumulation and diffusion

ICW propagation from an activated to an unactivated astrocyte relies both on GJC–mediated intercellular IP_3_ transport and on IP3 accumulation in the destination cell up to a threshold concentration that triggers CICR therein, thus locally regenerating the Ca^2+^ wave (Goldberg et al., 2010). The above analysis revealed that the most significant obstacle to ICW propagation is IP_3_ dilution to unactivated astrocytes. Indeed, when an astrocyte activates, the excess intracellular IP_3_ diffuses to every unactivated cell it is coupled to. However, the total amount of IP_3_ that is distributed by an activated astrocyte does not depend on the number of cells to which it is coupled. Therefore, the larger the number of cells coupled to an activated astrocyte (i.e., the larger its degree *k*), the smaller the amount of IP_3_ that diffuses to every individual unactivated cell. As a result, when IP_3_ flows from a source (activated) astrocyte to a destination (unactivated) one, the probability that the destination astrocyte effectively gets activated decreases when the source astrocyte is GJC-coupled to many unactivated cells.

**Figure 5:**
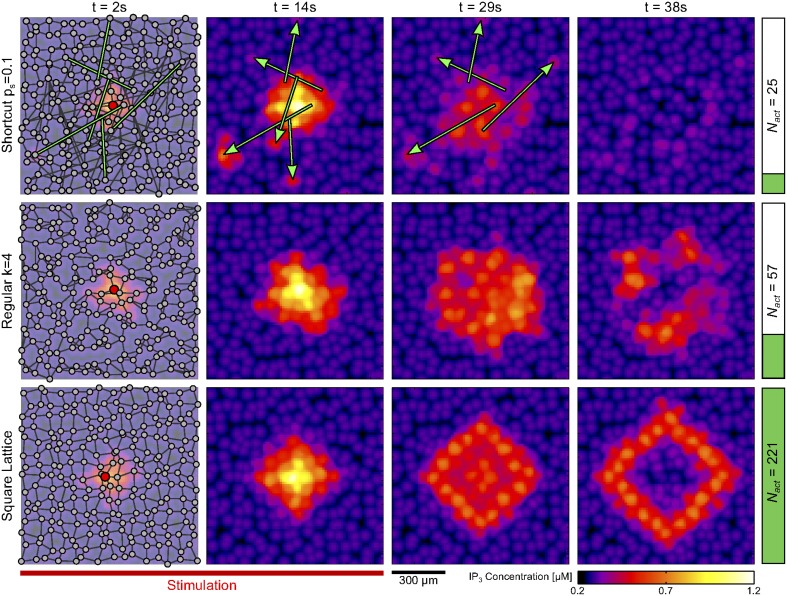
**Effect of long-distance GJC-coupling on ICW propagation**. Snapshots of IP_3_ concentration (see Supplementary Material S1.4) at different time instants in three 2D networks (shown in the *leftmost panels*): a shortcut network with *p*_*s*_ = 0.1 (*top row*); a regular network with 〈*k*〉 = 4 (*middle row*); and a square lattice also with 〈*k*〉 = 4 (*bottom row*). The presence of long-distance connections (*green edges*) in the shortcut network causes IP_3_ transport away from the wave front, hampering ICW propagation. This is reflected by a considerably lower value of the number of cells activated by the ICW (*N*_act_, *right vertical bars*) in the shortcut network compared to the other two networks. The ICW was triggered by stimulating the astrocyte marked in *red* (*leftmost panels*) from 0 s to 25 s (*red bar*). Astrocyte parameters as in Table 1.

This observation accounts for the simulation results of Figure 3A, where the propagation range decreases when the mean degree 〈*k*〉 of the GJC network increases. It also explains why long–distance connections between cells tend to hamper ICW propagation (see Supplementary Material S3). Figure 5 illustrates ICW propagation in time for three networks with identical mean degrees but different organization of the GJC couplings: a shortcut network (*top* panels), a regular network (*middle* panels) and a square lattice (*bottom* panels). Note that all the analyses and quantifications in this study were obtained with 3-dimensional networks, except in this figure where we show 2-dimensional networks for reasons of readability. The stimulation of an astrocyte in the center of the network (*red circles* in the leftmost panels) triggers an ICW that propagates toward the periphery. Whereas the ICW keeps on propagating long after the end of stimulation (at *t* = 25 s, *red bar*), both in the regular network and in the square lattice, it abruptly aborts (at *t* = 29 s) in the shortcut network. When IP_3_ is transported along a long–distance coupling, the destination astrocyte is very likely located far away from the propagation front, in a zone where most astrocytes are unactivated. It is thus likely that most of the astrocytes coupled to the destination astrocyte are unactivated, so that even though the destination astrocyte eventually gets activated, it cannot propagate the ICW further. This dilution effect, that hampers propagation, is much more reduced in networks with only short-distance couplings. Indeed, the destination cell of a short–distance GJC-coupling is by definition located in the vicinity of the propagation front and therefore is likely to be GJC-coupled to several activated astrocytes.

**Figure 6:**
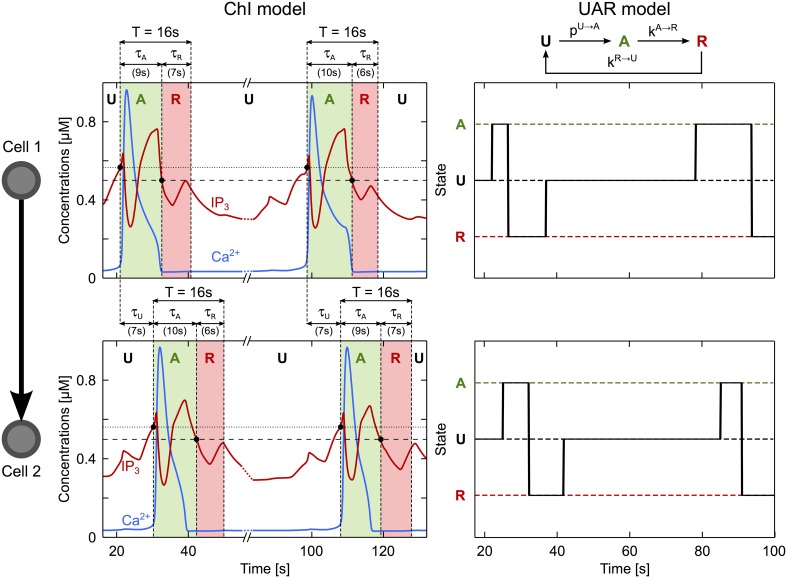
**A simplified description of ICW propagation**. The propagation of an ICW through an astrocyte may be regarded as a three-state process, as illustrated here for the case of two connected astrocytes (cell 1, *top panel*; cell 2, *bottom panel*). These astrocytes are in the unactivated state (U) when at rest. Upon arrival of an ICW (when the IP_3_ level crosses the threshold for CICR initiation, *dotted line*), the transient increase of intracellular Ca^2+^ (*blue traces*) occurring first in cell 1 then in cell 2, activates these cells (A, *green regions*). Following activation, each cell recovers to rest through a refractory period (R) when their IP_3_ value falls below the threshold gradient for intercellular diffusion (*dashed line*). The time constant of each transition may be estimated accordingly. τ_*U*_ coincides with the delay between the Ca^2+^ increases in cell 1 and in cell 2. τ_*A*_ is estimated by the time interval from the beginning of the Ca^2+^ elevation to the point where IP_3_ gets below the diffusion threshold. Finally, τ_*R*_ is estimated by τ_*A*_ + τ_*R*_ = *T* where *T* (= 16 s in this example) represents the minimum period of Ca^2+^ oscillations in a single cell. Astrocyte parameters as in Table 1.

The above rules can be expressed in a concise way by a simplified model of ICW propagation. To build this simplified model, we forget about the complex dynamics and interactions between Ca^2+^, IP_3_ and calcium stores that take place inside each astrocyte. Instead, we consider that an astrocyte is characterized by a single signaling state that can have only three values: (U)nactivated, (A)ctivated or (R)efractory (see Figure 6). The default (basal) state is the (U)nactivated one. When the ICW reaches astrocyte number *i, i* can become (A)ctivated with probability *p*^*U→A*^_*i*_ (that we define below). Once in the A state, the astrocyte first switches to the (R)refractory state with rate *k*^*A→R*^, then back to the U state, with rate *k*^*R→U*^.

At every time step, the model computes the propagation efficiency of each astrocyte according to the above mentioned rule: (*i*) an astrocyte can propagate the ICW only if it is activated by the ICW, and (*ii*) its efficiency to propagate the ICW decreases when the number of unactivated astrocytes to which it is GJC-coupled increases. Therefore, the propagation efficiency of astrocyte number *i*, β_*i*_, is computed according to

(7) $$\beta_{i}(t)=\{\begin{array}{lll} 1/N_{i}^{\text{u}}(t) & \text{if} & i\text{ is in the activated state at time }t\\ 0 & \text{otherwise} & \end{array}$$

where *N*^*u*^_*i*_(*t*) is the number of astrocytes that are GJC-coupled to *i* and are not in the activated state.

Now, to determine whether an astrocyte *i*, which is in the *U* state at time *t* will be activated by the ICW at time *t* + 1, the model computes the sum of the propagation efficiencies of all the astrocytes that are coupled to *i*, ∑_*j*∈
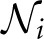
_*i*__ β_*j*_(*t*) (with 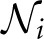
_*i*_ the set of astrocytes that are GJC-coupled to *i*). If this sum is larger than a threshold ϑ_*i*_, *i* gets activated (i.e., switch from *U* to *A* states) with rate *k*^*U→A*^. If the sum of efficiencies is not larger than ϑ_*i*_, the astrocyte remains in the *U* state. Formally, we thus define the global probability that an unactivated astrocyte *i* gets activated by the ICW as:

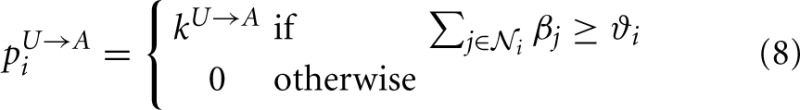



In the model, the threshold ϑ_*i*_ increases linearly with the astrocyte degree *k*_*i*_ (the number of cells it is coupled to), that is ϑ_*i*_ = *a* · *k*_*i*_ + *b* where the constants *a* and *b* where estimated from the *ChI* model (Supplementary Material S3 and Figure S5B). The other parameters of the simplified model, i.e., the rates *k*^*U→A*^, *k*^*A→R*^ and *k*^*R→U*^ were estimated from simulations of the *ChI* model as, respectively, the inverse of the time needed to transmit ICW between two cells (τ_*U*_), and the inverse of the time spent in the activated (τ_*A*_) or refractory (τ_*R*_) states (see Figure 6, parameter values reported in Supplementary Table S1).

The three-state UAR description introduced above is reminiscent of Susceptible–Excited–Refractory (SER) models widely adopted to study network signal propagation (Dodds and Watts, 2004; Müller-Linow et al., 2006; Centola et al., 2007; Müller-Linow et al., 2008; Hütt et al., 2012) except that our definition of *p*^*U→A*^_*i*_ takes into account the two-hop neighborhood of each astrocytes, i.e., the activation state of neighbors of the cells coupled to each astrocyte. If the status of activation of the two-hop neighborhood is indeed crucial in ICW propagation, then we expect that the essence of ICW dynamics in the astrocyte networks considered so far, will be reproduced if we substitute the *ChI* astrocyte model by the UAR description. To test this, we simulated 3D networks with the same topologies than those in Figure 3 except that now, the internal dynamics inside each node are given by the simple stochastic UAR model.

**Figure 7:**
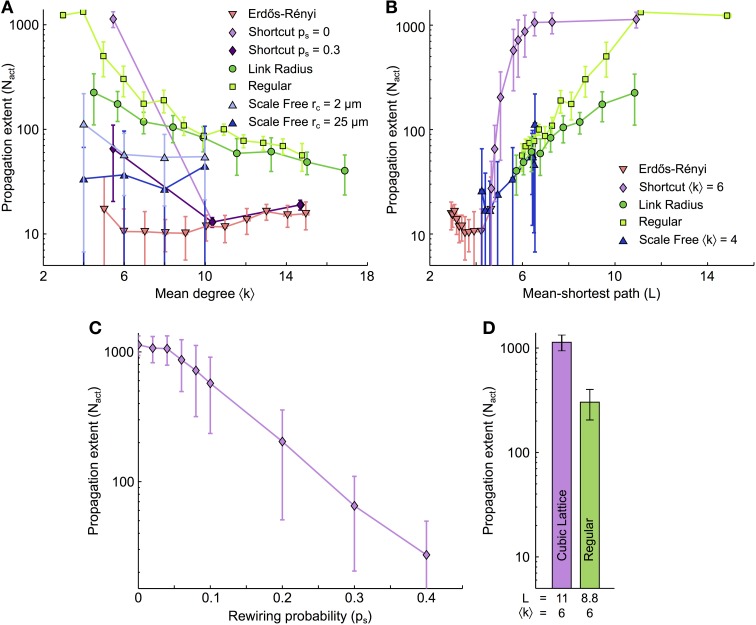
**ICW propagation simulated in the simplified model**. **(A)** The extent of ICW propagation (*N*_act_) as a function of 〈*k*〉 and **(B)** *L* was recomputed for the same coupling networks as in Figure 3 but with astrocytes that were modeled with the simplified three–state UAR model. The similarity of *N*_act_ values thus obtained compared to those obtained in Figure 3 with the *ChI* model provides the simplified model with biophysical consistency. This supports our statement that the activation state of the two–hop neighborhood of individual cells (“the cells that are connected to the cells that are connected to me”) is crucial to ICW propagation. **(C)** In shortcut networks, a large density of long–distance couplings between astrocytes hampers ICW propagation, like with the *ChI* model in Figure 3. **(D)** Propagation extent for cubic lattices and regular networks of same mean degree. Data points ± errorbars correspond to mean values ± standard deviation over 20 networks of similar topology. Parameters of the simplified model (see text) as in Supplementary Table S1 (τ values were estimated like in Figure 6).

Figure 7 shows the extent of ICW propagation simulated by the simplified UAR model in the same networks as those considered in Figure 3. It is apparent that the UAR model produces a good qualitative match of the results on ICW propagation previously obtained by the *ChI* model (Figure 3). The number of activated cells (*N*_act_) indeed decreases for either large values of the mean degree (〈*k*〉, Figure 7A) or small values of the mean shortest path (*L*, Figure 7B). As in Figure 3, the dependence on the mean shortest path *L* shows two distinct groups: the propagation ranges of all the coupling organizations where the GJC couplings are strictly restricted to short distance collapse roughly to the same curve, whereas the networks with long-range GJC couplings form another group. As with the detailed *ChI* model, the propagation range on scale-free networks crossovers from the behavior typical of long-distance couplings to that observed for organizations with short-distances couplings only. Data analysis also shows a comparable decrease of the propagation extent when the fraction of long-distance GJC couplings (*p*_*s*_) increases (Figure 7C). Remarkably, the UAR description even reproduces the distinct propagation extents between cubic lattices and regular networks (Figure 7D).

To conclude, these results confirm that the basic ingredients expressed in the UAR model are sufficient to explain the propagation extent of ICW based on the full bio-realistic *ChI* model. This confirms that the probability for an astrocyte to propagate an incoming ICW does not only depend on the number of activated cells to which it is coupled (i.e., its 1-hop neighbors), but also on its 2-hop neighborhood, i.e., the cells that are coupled to the cells coupled to this astrocyte.

### The influence of the coupling strength is non–monotonous

Thus far, our simulations consisted in changing the topology of the GJC network, keeping GJC strength (or conductance) constant and identical for all cell-cell couplings and all stimulations. In this final section, we turn to estimate how ICW propagation range changes when the overall coupling strength varies. We used *Link Radius* networks for the coupling topology since the properties of this topology (spatially constrained and distributed degrees) are likely closest to real astrocyte networks. All the other simulation parameters were kept unchanged compared to the above results.

**Figure 8:**
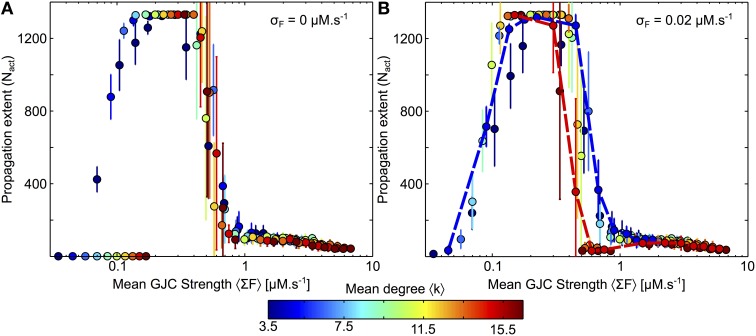
**Changes of GJC strength and mean degree in Link Radius networks**. **(A)** In a first approach the strength *F* of the GJC couplings between two coupled cells are identical for every coupled cell pairs. Whatever the mean degree of the network, the ICW extent is dictated by the mean GJC strength per node 〈Σ*F*〉 = 〈*k*〉 *F*. **(B)** When the GJC strengths are randomly chosen from a normal distribution, the dependance on 〈Σ*F*〉 is essentially preserved, except that the optimal range of 〈Σ*F*〉 values diminishes when the network mean degree is large (*dashed red lines*). All points represent 20 realization of a given parameter combination. Curves and points are color-coded according to the mean degree 〈*k*〉. Astrocyte parameters as in Table 1 except for *F* that was varied between 0 and 0.05 by steps of 0.01 and between 0.1 and 0.4 by steps of 0.05. Link Radius parameters as in Table 2.

We first changed the strength *F* of all GJC. In order to compare the resulting networks where both the mean degree 〈*k*〉 and the coupling strength per connection, *F*, vary, we used the mean GJC strength per cell 〈Σ*F*〉 = 〈*k*〉*F*. As shown in Figure 8, this quantity was found to be a very good predictor of ICW extent. Figure 8A illustrates the propagation range for the case where the coupling strength is the same for every coupled astrocyte pairs. Whatever the mean degree 〈*k*〉, these results show a non-monotonous behavior of the ICW propagation, with optimal ICW propagation at intermediate coupling strengths. Actually, large coupling strengths (i.e., 〈Σ*F*〉 > 1 μM.s^−1^) severely hinder propagation, in agreement with our conclusions above with constant *F* values and increasing mean degrees 〈*k*〉. For very low values of *F* (〈Σ*F*〉 < 0.1 μM.s^−1^), ICW propagation is as well blocked because IP_3_ diffusion is much slower than its degradation. In between those two regimes (i.e., for 0.1 <〈Σ*F*〉 < 1 μM.s^−1^), ICW propagation is optimal and actually reaches the whole astrocyte network (regenerative propagation). Therefore, one expects from these data that ICW propagation should be optimal in networks with intermediate coupling strength since large and small strengths both hinder it. Note however that the regenerative propagation observed at intermediate values of 〈Σ*F*〉 is qualitatively different from the propagation observed at large values (and in the simulations presented in the article thus far). With such intermediate values, the activation of a cell by a Ca^2+^ wave switches the cell to a “Up” state, with much larger values of IP_3_ and Ca^2+^ than before the activation (Figure S11A). The biological relevance of this “Up” state is not firmly ascertained yet.

Finally, we investigated the effect of randomly distributed GJC strength on ICW propagation. To this end, we picked each GJC strength independently according to a normal distribution with mean *F* and variance σ^2^_*F*_. To avoid negative values and preserve constant mean, we restricted the random values to [0, 2*F*]. Figure 8B shows that this distribution of the GJC over cell pairs does not substantially affect ICW propagation. The only notable change is seen for high-degree networks (*red dashed curve*) for which the end of the optimal (regenerative) regime occurs earlier, i.e., for smaller GJC strengths. The dependence on the network mean degree is amplified when the variability of the GJC strength increases (Figure S11B): the variability of the GJC strength hardly has an effect on low-degree networks, but tends to hinder ICW propagation in high-degree networks. Having low connectivity is thus beneficial for ICW propagation in two ways: (1) it increases ICW extent, for all GJC strength *F*; (2) it makes the network more robust to variability in GJC strengths.

## Discussion

In many instances, the variability observed in the propagation range of intercellular calcium waves (ICW) in astrocyte populations (Charles, 1998; Scemes and Giaume, 2006; Kuga et al., 2011; Sasaki et al., 2011) cannot be accounted for by the type of preparation or by stimulation protocol (Scemes and Giaume, 2006). Our model of ICW in 3D coupling networks suggests that this variability can be due to a mere change in the spatial organization (or topology) of the astrocyte network. In our simulations, the mean degree 〈*k*〉 and mean-shortest path *L* of the coupling networks were found to be the main topological characteristics controlling propagation. Quite surprisingly, increasing the number of cells coupled to each astrocyte or adding long–distance GJC couplings actually reduced the extent of ICW propagation. Moreover, all types of ICW propagations ranges could be reproduced in our model with spatially constrained networks (Link Radius and Regular) just by changing 〈*k*〉. Our model therefore predicts that variations in the organization of the GJC couplings control the range of ICW propagation.

A first experimental element in favor of our hypothesis is the observation that regenerative ICW are far more frequent in cell cultures than in slices or *in vivo* experiments (Scemes and Giaume, 2006). Since a 2D embedding imposes a lower mean degree (compared to 3D), this observation supports our hypothesis. *In vivo*, the organization of astrocyte coupling networks has recently attracted attention as several articles demonstrated variability between brain regions (Giaume et al., 2010). For instance, heterogeneities in coupling organization were found in mouse olfactory glomeruli (Roux et al., 2011), and somatosensory cortex (Houades et al., 2008). Locally, astrocyte density may control the astrocyte coupling organization, as in the stratum pyramidale of the hippocampus (Rouach et al., 2008). These local heterogeneities are also reflected in the observation that the total number of coupled astrocytes (obtained via e.g., biocytin-coupling experiments) vary a lot between brain regions: cortical astrocytes can be organized in networks of hundreds of cells (Nimmerjahn et al., 2004) while in the hippocampus, astrocytes in the CA3 region are much less coupled than in CA1 (D'Ambrosio et al., 1998). These variations in coupling can also be at least partially attributed to variations in the expression of connexins (Cx) 43 and 30, which also displays high heterogeneities (Giaume and Theis, 2010); for instance, hypothalamus and hippocampus display higher Cx43 levels than cortex and brain stem astrocytes (Blomstrand et al., 1999). According to our hypothesis, this regional variability of the organization of the astrocyte coupling network could explain the regional variability of the extent of ICW propagations.

While the heterogeneity of the coupling organization is being increasingly recognized (Giaume et al., 2010), only a few studies have addressed the relationship between the coupling properties of the astrocytes (or their Cx expression) and ICW propagation. According to our hypothesis, in regions in which GJC intercellular communication is the main ICW pathway (retina (Newman, 2001), striatum (Venance et al., 1997) and cerebral cortex (Iwabuchi et al., 2002; Haas et al., 2006)), one should observe increased ICW extent when the astrocytes are less coupled, or when Cx expression is lower. Blomstrand et al. (1999) quantified both the extent of dye coupling and ICW propagation as well as Cx43 expression in astrocyte cultures from different brain regions. In accordance with our hypothesis, an inverse relationship between ICW extent and GJC coupling was reported for two brain regions: hypothalamus was found to be highly GJC-coupled and to support small extent ICW whereas the neocortex, that was less GJC-coupled, exhibited larger ICW (Blomstrand et al., 1999). More generally, GJC coupling in regions where intercellular GJC is the predominant pathway for ICW propagation is often reported to be lower than in other brain regions like in the striatum (Rouach et al., 2002) and cortex (Aberg et al., 1999; Blomstrand et al., 1999). In the CA3 region of the hippocampus, known to be less coupled than CA1 (D'Ambrosio et al., 1998), neuronal activity is able to trigger long range ICW (Dani et al., 1992). On the contrary, increased coupling induced by forced expression of Cx43 was found to decrease ICW extent in human 1321N1 astrocytoma cells (Suadicani et al., 2004). Taken together, these articles comfirm our hypothesis: highly coupled astrocyte networks display small extent ICW while less coupled ones display larger ICW.

If astrocytes are indeed organized in independent non–overlapping domains (Bushong et al., 2002), the organization of their coupling can be expected to be close to a Voronoi diagram of the cell centers (Aurenhammer, 1991). The mean degree of a Voronoi diagram in three dimensions is ≈ 15, a value that should prevent ICW propagation according to our simulation results (see Figure 3A for 〈*k*〉 = 15). Actual astrocyte networks however differ from a pure Voronoi diagram because some of the astrocytes can be disconnected from the GJC network (Theis and Giaume, 2012). For instance, in cocultures of rat striatal neurons and astrocytes, 21% of the astrocytes were found to be disconnected from the network (Rouach et al., 2000). This figure even increases to 40% of disconnection in cultures with only astrocytes. Removing 21 or 40% of the nodes from a Voronoi diagram leads to a mean degree 〈*k*〉 ≈ 11.7 or 8.9, respectivley. Interestingly, these values of the mean degree are close to values reported *in situ*: 〈*k*〉 ≈ 11 neighbors in CA1 rat hippocampus (Xu et al., 2010). Our simulations show a strong increase in the ICW propagation range when the mean degree becomes smaller than ≈ 8 − 10 (Figure 3A). Interestingly, both Cx30 and Cx43 expression and permeability can be regulated by neurons (Rouach et al., 2000; Koulakoff et al., 2008; Roux et al., 2011), possibly via extracellular K^+^ (Pina-Benabou et al., 2001). This K^+^-triggered increase in GJC communication was also recently reported to decrease ICW extent in astrocyte networks [see Figure 3 in Scemes and Spray (2012)], in accordance with our hypothesis. Therefore, neurons could modulate mean degree of the astrocyte coupling network and even trigger a switch between 〈*k*〉 ≈ 12 and 〈*k*〉 ≈ 8 thus allowing the propagation of ICW to long ranges.

The coupling organization in astrocyte networks also changes during development. During the first postnatal weeks, astrocytes show large increases in Cx43 expression that persists until adulthood (Aberg et al., 1999; Montoro and Yuste, 2004). ICW are frequently observed during development (Parri et al., 2001; Weissman et al., 2004; Fiacco and McCarthy, 2006; Scemes and Giaume, 2006; Kunze et al., 2009) and are thought to be much less frequent in adults under non–pathological conditions (Fiacco and McCarthy, 2006; Scemes and Giaume, 2006). In most parts of the brain, Cx43 becomes strongly expressed between postnatal day 10 (Aberg et al., 1999) and the third postnatal week (Rouach et al., 2002). In agreement with our hypothesis, spontaneous astrocyte calcium activity in the rat ventrobasal thalamus decreases by five fold between postanatal day 10 and 14 (Parri et al., 2001). In mice CA1 stratum radiatum in the hippocampus, realistic stimulation with caged IP_3_ failed to elicit ICW in individuals ranging from postnatal day 10–14 (Fiacco and McCarthy, 2004). The astrocytes of the neocortex are known to be sparsely coupled during postnatal days 1–3 (Aberg et al., 1999), where ICW were found to be propagating (Iwabuchi et al., 2002). On the opposite, in the CA1 region of the hippocampus, astrocytes are highly coupled during postnatal day 10–14 (Aberg et al., 1999) and they do not support wave propagation with similar stimulation protocols (Fiacco and McCarthy, 2004). Taken together, these observations support our hypothesis, since ICW activity seems to be predominant during early postnatal development when astrocytes are less coupled, and tends to diminish with age as Cx expression gets stronger.

In several regions of the brain, intercellular IP_3_ transfer through GJC coupling is however not the major pathway for ICW. Indeed, ICW propagation through the activation of P2Y receptors by diffusing extracellular ATP is the dominant pathway in the corpus callosum (Schipke et al., 2002), CA1 hippocampus (Haas et al., 2006) and the cerebellum (Hoogland et al., 2009). In these regions, ICW can be elicited through ATP application and are strongly diminished by P2Y receptor antagonists (Hoogland et al., 2009; Kuga et al., 2011). ATP activation of P2Y1 receptors leads to IP_3_ production via PLCβ activation but ATP also has a P2Y-mediated effect on GJC permeability, that was reported to decrease gap junction coupling (Rouach et al., 2002; Même et al., 2004; Orellana et al., 2013). A network with initially strong GJC-coupling would not support long range ICW propagation via the intercellular GJC pathway according to our model. However, the release of ATP in such a network, by down-regulating GJC permeability, would decrease the coupling and facilitate ICW propagation. In support of this hypothesis, the expression of P2Y1R in Cx43 expressing cells increases ICW extent while the expression of Cx43 in the absence of P2Y1 reduced it (Suadicani et al., 2004). Collectively, these articles also support our hypothesis and offer an attractive perspective: long-range ICW can be elicited in an astrocyte network (*i*) if its mean degree of GJC coupling is low - for regions in which GJC is the predominant pathway; or (*ii*) if the mean degree is large, for regions in which ATP is the predominant pathway, but if propagation is rescued by the down-regulation of GJC permeability by ATP.

The topological determinants of signal propagation in astrocyte networks thus seem different from those at play in neuronal networks. Signal is propagated from one astrocyte to the other by diffusion from a single IP_3_ pool (the astrocyte) while neurons communicating through chemical synapses use distinct pools of neurotransmitters located in each of their synapses. This difference is actually the strongest one in our view: while increasing the number of neighbors in astrocyte networks dilutes away IP_3_ and decreases ICW propagation, increasing the number of neighbors in neuronal networks only implies the addition of new synapses and can thus only increase the network excitability. In agreement with this view, increased connectivity (i.e., mean degree) has been shown to promote synchronization in model networks of excitable neurons (Wang et al., 1995; Golomb and Hansel, 2000) and to control the switch between asynchronized states and partially synchronized (or coherent) states (Olmi et al., 2010; Luccioli et al., 2012; Tattini et al., 2012). In the present study, the presence of hubs and long range connections between astrocytes impaired ICW extent. In contrast, broad in-degree distributions (allowing the presence of hubs) has been shown to increase the mean activity in model neuronal networks while broad out-degree distributions increased the amplitude of cross-correlation in synaptic currents (Roxin, 2011). Finally, Dyhrfjeld-Johnsen et al. (2007) showed that network hyperexcitability during simulated sclerosis can be directly linked to the presence of long distance links in the network: when these long distance links were removed during maximal sclerosis, network hyperexcitability decreased. Theoretical studies suggested that to keep network activity balanced, synaptic weights of each neuron should be rescaled by some function of the neuron degree (Van Vreeswijk and Sompolinsky, 1996, 1998; Lerchner et al., 2006). This *synaptic scaling* mechanism has mainly been observed experimentally for post-synaptic terminals (Turrigiano, 2008). In astrocyte networks, one could wonder whether such a mechanism would influence ICW propagation. Unfortunately, because of the bidirectionality of these networks, rescaling GJC strength is not as straightforward as for neuronal networks (conductances in both direction should be equal *g*_*ij*_ = *g*_*ji*_ but *k*_*i*_ can be different from *k*_*j*_). Neuronal networks can also be bidirectional when neurons are coupled by GJC-mediated electrical synapses. While being restricted to certain adult neuronal subpopulations (Söhl et al., 2005), these GJC are still functionally relevant and have been shown to mediate synchronization between neurons both experimentally (Connors and Long, 2004) and in modeling studies (Chow and Kopell, 2000; Lewis and Rinzel, 2003). The effect of GJC topological properties such as connectivity and rewiring on signal propagation could however be more subtle. Because of the similarity between the term governing the diffusion of IP_3_ between astrocytes (*F* × (*I*_*i*_ − *I*_*j*_)) and the term governing GJC-coupling at electrical synapses (*g* × (*V*_*i*_ − *V*_*j*_)), GJC-coupled neuronal networks are of particular relevance to our study. Accordingly, the effects of network topology on signal propagation in GJC-coupled neuronal networks bear some resemblance with the effects described here for astrocyte networks. In Volman et al. (2011), it is shown that increasing GJC conductance or connectivity could help reduce epileptic seizures by mechanisms similar to what we observed in astrocyte networks (subthreshold activity gets diluted among neighbors). Further increases in connectivity however enhanced seizure activity (and thus signal propagation), in contrast with what we observed in astrocyte networks. Increased rewiring of GJC-coupled model neurons has been shown to impair signal detection but, differently from astrocyte networks, weak connectivity and coupling, while enhancing signal detection, impaired signal propagation (Volman and Perc, 2010). Altogether, those behaviors reported for neuronal networks do not match the observations we reported here for model astrocyte networks. These differences in the dynamics-topology relationships bring new light on the well-known observation that neurons form highly connected networks with long distance links while astrocytes usually restrict their couplings to a handful of nearby neighbors (Bushong et al., 2002). The topology of each of these cellular networks thus appears adapted for optimal signal transmission.

## Fundings

This study was funded by the High Council for Scientific and Technological Cooperation between France and Israel (Jules Lallouette, Hugues Berry), by the ERCIM “Alain Bensoussan” Fellowship (Maurizio De Pittà), and by the Italian–Israeli Joint Neuroscience Lab (Eshel Ben-Jacob, Maurizio De Pittà). The authors also acknowledge the support of the computing center IN2P3 at CNRS (http://cc.in2p3.fr), where the simulations were performed.

### Conflict of interest statement

The authors declare that the research was conducted in the absence of any commercial or financial relationships that could be construed as a potential conflict of interest.
