# Supplementary material for: Sparse short-distance connections enhance calcium wave propagation in a 3D model of astrocyte networks
Source: Front Comput Neurosci. 2014 Apr 16;8:45. doi: 10.3389/fncom.2014.00045 (PMC3997029; doi:10.3389/fncom.2014.00045)
Supplement: Supplementary file 1 [file Presentation1.PDF]

SUPPLEMENTARY MATERIAL

Sparse short-distance connections enhance calcium wave propagation in a 3D model of astrocyte networks

Jules Lallouette[1,2], Maurizio De Pittà[1,2,3], Eshel Ben-Jacob[3,4], Hugues Berry[1,2*]
1 EPI Beagle, INRIA Rhône-Alpes, F-69603, Villeurbanne, France
2 LIRIS, Université de Lyon, UMR 5205 CNRS-INSA, F-69621, Villeurbanne, France
3 School of Physics and Astronomy, Tel Aviv University, Ramat Aviv, Israel
4 Center for Theoretical Biological Physics, Rice University, Houston, TX, USA
$*$ **E-mail: hugues.berry@inria.fr**

S1 Astrocyte Ca^{2+} signalling

S1.1 The *ChI* model for astrocyte Ca^{2+} signalling

The possible signalling pathways underlying astrocyte Ca^{2+} signalling are not fully resolved. In general, astrocyte Ca^{2+} signalling relies on an intricate interplay of amplification, buffering, and extrusion pathways linked to cytosolic Ca^{2+} elevations that are mediated both by Ca^{2+} influx from the extracellular space into the cytoplasm [1, 2] and by Ca^{2+} release from the endoplasmic reticulum (ER) stores [3].

Inositol 1,4,5-trisphosphate-(IP_3-)triggered Ca^{2+}-induced Ca^{2+} release (CICR) from the ER is considered the primary mechanism responsible for intracellular Ca^{2+} dynamics in astrocytes [4, 5]. This mechanism, is essentially controlled by the interplay of three fluxes: (1) a Ca^{2+} uptake from the cytosol to the ER (J_P) that is mediated by (sarco)endoplasmic-reticulum Ca^{2+}-ATPase (SERCA) pumps, and keeps ER Ca^{2+} concentration at rest higher than in the cytosol; (2) a passive Ca^{2+} leak (J_L) from the ER to the cytosol that is driven by the Ca^{2+} gradient between them; and (3) an efflux (J_C) from the ER to the cytosol that is mediated by IP_3 receptor (IP_3R) channels and nonlinearly depends on both cytosolic IP_3 and Ca^{2+} [6].

Cytosolic Ca^{2+} regulates IP3Rs in a biphasic manner: Ca^{2+} release from the ER is potentiated at low cytosolic Ca^{2+} concentrations but is inhibited at higher Ca^{2+} concentrations [7, 8]. On the other hand, IP_3 monotonically activates IP3R channels at constant Ca^{2+} concentrations [9], but dynamically changes the Ca^{2+} sensitivity of the channel [6,10,11]. At low, subsaturating IP_3 concentrations, the optimal Ca^{2+} concentration for IP3R modulation becomes lower, whereas at high IP_3 concentrations, channel activity persists at supramicromolar Ca^{2+} concentrations [10,11]. Thus, the level of IP_3 determines the dynamics of intracellular Ca^{2+}.

Both production and degradation of IP_3 depend on enzymes that are regulated by cytosolic Ca^{2+} [12, 13]. These include Ca^{2+}-dependent IP_3 synthesis mediated by PLCδ (J_δ) and Ca^{2+}-dependent IP_3 degradation by IP_3-3K (J_{3K}) and by IP-5P (J_{5P}) [14–16]. Under proper assumptions however, the dependence of IP-5P on Ca^{2+} may be neglected and intracellular Ca^{2+} dynamics in an astrocyte may be well captured by a set of only three equations: one for Ca^{2+} (C), one for IP_3 (I) and the third one for the deinactivation probability of IP3R channels (h). These three equations constitute the so-called *ChI* model of intracellular Ca^{2+} dynamics in an astrocyte and read [17]:

$$\frac{\mathrm{d}}{\mathrm{d}t}C = J_C(C,h,I) + J_L(C) - J_P(C) \tag{S1}$$

$$\frac{\mathrm{d}}{\mathrm{d}t}h = \Omega_h(C,I) \cdot (h_\infty(C,I) - h) \tag{S2}$$

$$\frac{\mathrm{d}}{\mathrm{d}t}I = J_\delta(C,I) - J_{3K}(C,I) - J_{5P}(I) \tag{S3}$$

with:

$$J_C(C,h,I) = \Omega_C \cdot m_\infty^3 h^3 \cdot (C_T - (1+\rho_A)C)$$
$$J_L(C) = \Omega_L \cdot (C_T - (1+\rho_A)C)$$
$$h_\infty(C,I) = d_2 \frac{I+d_1}{d_2(I+d_1)+(I+d_3)C}$$
$$J_\delta(C,I) = O_\delta \cdot \frac{\kappa_\delta}{\kappa_\delta + I}\mathcal{H}\left(C^2, K_\delta\right)$$
$$J_{5P}(I) = \Omega_{5P} \cdot I$$
$$m_\infty(C,I) = \mathcal{H}\left(C, d_5\right)\mathcal{H}\left(I, d_1\right)$$
$$J_P(C) = O_P \mathcal{H}\left(C^2, K_P\right)$$
$$\Omega_h(C,I) = \frac{O_2 d_2(I+d_1) + O_2(I+d_3)C}{I+d_3}$$
$$J_{3K}(C) = O_{3K} \cdot \mathcal{H}\left(C^4, K_D\right)\mathcal{H}\left(I, K_3\right)$$

where the function $\mathcal{H}\left(x^n, K\right)$ denotes the sigmoid (Hill) function $\frac{x^n}{x^n+K^n}$. The values of the parameters in the above equations are given in Table 1.

S1.2 Intercellular IP_3 diffusion

Astrocytic Ca^{2+} signals can propagate by at least two routes [18]: one is intracellular, by diffusion of IP_3 directly from cytoplasm to cytoplasm via gap junction channels (GJCs); the other is extracellular, by release of ATP from astrocytes which binds to G-protein coupled receptors (GPCR) on the same cell or neighboring ones, increasing IP_3 and triggering CICR therein [19]. Although these two routes need not be mutually exclusive and their relative contributions likely depend on developmental, regional and physiological states [18,20,21], experiments suggest that GJC–mediated intercellular propagation is likely the predominant signalling pathway in many astrocyte types [22, 23] and accordingly, in this study we consider only the latter.

From a modelling perspective, IP_3 diffusion from one astrocyte i to a neighboring one j, accounts for an additional term (J_{ij}) in equation (S3) which, in the most general form, can be thought as some function ϕ of the IP_3 concentration gradient between cells i and j, i.e. $\Delta_{ij}I = I_i - I_j$, so that [24]:

$$J_{ij} = \phi(\Delta_{ij}I)\ . \tag{S4}$$

In the simplest scenario, ϕ may be assumed linear [25, 26], and J_{ij} be described accordingly by Fick's first diffusion law, so that

$$J_{ij} = -F_{ij} \cdot \Delta_{ij}I \tag{S5}$$

where F_{ij} is the diffusion coefficient. Moreover, when considering intercellular propagation of global/whole–cell Ca^{2+} signals, IP_3 diffusion from one cell soma to another has to be considered and equation (S5) might not be valid for this case. As illustrated in Figure S1A in fact, connections between astrocytes through GJCs are mostly at the cell distal processes [21] whose complex morphology and narrow intracellular space [27, 28] could considerably hinder IP_3 diffusion from/to somata. Moreover, GJCs cluster at discrete sites of these processes [29], thereby constraining the diffusion pathway of IP_3 from one cell to another. Finally, IP_3 production and degradation in the processes could either promote IP_3 transfer between cells or hamper it. In this fashion, the ensemble of astrocytic processes and GJCs interposed between cell somata (i.e. the region between the *dashed lines* in Figure S1A) could be equivalently regarded as a diffusion barrier for IP_3 exchange between cells, and accordingly, IP_3 diffusion between cells could be inherently nonlinear. This scenario is further substantiated by growing experimental evidence suggesting that GJC permeability could be actively modulated by various factors, including different second messengers [30]. With this regard, the permeability of Cx43, a predominant connexin in astrocytic GJCs [29], could be modulated for example by phosphorylation by PKC [31, 32]. Because the same kinase also takes part in IP_3 degradation as well as in Ca^{2+} signalling, this possibility ultimately hints that GJC permeability could also depend on IP_3 signalling whose dynamics is notoriously nonlinear [13, 17].

Thus, together with linear diffusion (equation (S5)), we consider an alternative expression for J_{ij} to

account for nonlinear IP_3 diffusion too. In particular we assume that IP_3 diffusion between two contiguous astrocytes, i and j, is a threshold function of the IP_3 gradient ideally measured across processes and GJCs in the cell somata (i.e. above and below the *dashed lines* in Figure S1A), and is limited by the maximal GJC permeability. Accordingly, a possible expression for J_{ij} reads [33]:

$$J_{ij} = -\frac{F}{2}\left(1 + \tanh\left(\frac{|\Delta_{ij}I| - I_\theta}{\omega_I}\right)\right)\frac{\Delta_{ij}I}{|\Delta_{ij}I|}\,. \tag{S6}$$

Figure S1B shows J_{ij} as a function of $\Delta_{ij}I$ in the linear vs. nonlinear approximation of IP_3 diffusion (*orange* vs. *black curves* respectively) where I_θ in equation (S6) is the threshold gradient for which effective IP_3 diffusion occurs, that is $J_{ij} > 0$ only if $|\Delta_{ij}I| > I_\theta$; whereas ω_I scales how fast J_{ij} increases (decreases) with $\Delta_{ij}I$ beyond this threshold. The parameter F sets the slope of J_{ij} in the linear approximation (equation (S5)), while in the nonlinear case, it corresponds to the maximal incoming (minimal outgoing) diffusion fluxes, and can be regarded as a measure of GJC permeability [33].

S1.3 Stimulation

To induce Ca^{2+} wave propagation, the central cell i in the network (hereafter dubbed as "driving" cell), is selectively stimulated by injection of a supplementary IP_3 flux $J_{0,i} = \phi(\Delta I_{0i})$ which is modeled by equation (S6) as a function of a biasing gradient $\Delta I_{0i} = I_{bias} - I_i$. In the latter, I_{bias} stands for a nonspecific IP_3 concentration bias which could be reproduced by different experimental conditions, such as for example by IP_3 production through GPCR activation by synaptic stimuli impinging on the driving cell [34, 35], or by IP_3 uncaging therein [5, 36]. With regards to the simulations in this study, I_{bias} was modeled by a rectangular pulse comprised between 0 and t_s, where t_s was fixed to allow Ca^{2+} waves to fully propagate to their maximum extent (Figure S2). In particular, in this study, the total number of activated cells in a simulation was found to be independent of t_s when $t_s \geq 100$ s for $I_{bias} \geq 2$ μM (see Figure S2). Accordingly, for a total simulated time T, $t_s = T$ was chosen.

This stimulation protocol can be viewed as the modeling counterpart of the following stimulation protocols used in actual experiments:

- stimulation of one astrocyte with an IP3-filled pipette (equivalent to the dummy cell that we use);
- IP3 uncaging in a small area surrounding one astrocyte (because simulation in the model usually activates all the neighbors of the stimulated astrocyte);
- physical stimulation of a small area (for the same reasons).

In vivo, this could also be the result of increased synaptic activity in a small area which leads, through activation of m-GluR, to IP3 synthesis by PLC-beta (cf. [17]).

S1.4 ICW Visualization

Snapshots of ICW propagation in Figure 4 were obtained from simulations of 2D networks with the *ChI* model. Image resolution was first fixed (75×75 pixels there) and each pixel scaled from 0 to 1. At each time t, each pixel $P_{xy}(t)$ in column x and row y was then computed using:

$$P_{xy}(t) = \frac{(\kappa_{xy}(t) - \chi_{xy}) - \min_{i,t} I_i(t)}{\max\limits_{i,t} I_i(t) - \min\limits_{i,t} I_i(t)} \tag{S7}$$

where $I_i(t)$ is the IP_3 concentration of astrocyte i at time t. The term $\kappa_{xy}(t)$ is the contribution of signals to the pixel (x, y) while χ_{xy} is a term added to improve contrast with extra-cellular spaces:

$$\kappa_{xy}(t) = \frac{\sum\limits_{i=0}^{N} I_i(t) e^{-\alpha||\overrightarrow{c_i} - \overrightarrow{c_{xy}^{img}}||}}{\sum\limits_{i=0}^{N} e^{-\alpha||\overrightarrow{c_i} - \overrightarrow{c_{xy}^{img}}||}} \tag{S8}$$

$$\chi_{xy} = \frac{\gamma}{2}\left(\tanh\left(\frac{\min_i ||\overrightarrow{c_i} - \overrightarrow{c_{xy}^{img}}|| - \beta}{\beta}\right) + 1\right) \quad \text{(S9)}$$

where N is the number of astrocytes; $\overrightarrow{c_i}$ is the position of cell i; $\overrightarrow{c_{xy}^{img}}$ is the rescaled position of the pixel in μm; α is a rescaling factor; β is the default length of intercellular space; and γ determines the intensity of the darkening of extracellular space. In this study: $\alpha = 10\ \mu\text{m}^{-1}$; $\beta = 35\ \mu$m and $\gamma = 4$. Finally, during the rendering process if P_{xy} was found below 0 or above 1, it was set to 0 or 1 respectively.

S2 Dependence of ICW propagation on network topology

To identify which topological features could account for the differences in ICW propagation, the extent of ICW propagation in networks of different topology was studied as a function of four main topological measures: mean degree ($\langle k \rangle$), mean shortest path (L), mean clustering coefficient ($\langle C \rangle$), and mean hierarchical clustering coefficient ($\langle cc_d \rangle$ with $d = 3$) [37–39]. Comparison of the results for $\langle k \rangle$ and L in Figure 3 in the text with those for the clustering coefficients in Figure S3, revealed that the latter two characteristics could not generally account for differences in the ICW propagation. Values of N_{act} vs. $\langle C \rangle$ as well as of N_{act} vs. $\langle cc_d \rangle$ were found indeed either to be scattered considerably more than those for $\langle k \rangle$ and L (Figure S3A), or to cluster together, regardless of network topology (Figure S3B). These results are robust, as data points are reported as mean values±std on 20 different realizations of each network with the same topology and clustering coefficient. Accordingly, only $\langle k \rangle$ and L were considered in this study as pertinent measures to describe the topological features that could critically regulate ICW propagation.

S3 Supplementary Analysis

S3.1 Local ICW propagation

The biophysical mechanisms that control the extent of ICW propagation in astrocyte networks may be revealed by considering ICW propagation locally: that is, at the level of small ensembles of neighboring connected cells of the network. Figure S4A for example, shows a small portion of a larger astrocyte network, where a Ca^{2+} wave coming from the cells in the upper left corner (*olive green squares*) is propagating to cells **A** and **B** (*green squares*). In presence of sufficient IP_3 diffusion out of these cells, this wave would continue to cells **C** and **D** in the next step of propagation (*red arrows*) and later on to all those cells pointed by the *blue arrows*. Yet, because **C** is connected to two unactivated cells while **D** only to one, then IP_3 must diffuse out of **C** twice faster than out of **D**, making it harder to reach the CICR threshold in **C** and thus reducing the chances that the wave could propagate through it. By similar arguments, because **B** is connected to three unactivated cells, whereas **A** only to two, IP_3 supply to those cells from **B** is less than from **A**, so that the chances that a cell could get activated by **B** are lower than by **A**, as reflected by line arrows of different width. These arguments thus hint that astrocytes that have a larger number of unactivated neighbors are in general, less likely to propagate Ca^{2+} waves essentially due to two mechanisms at play: (1) it is harder for intracellular IP3 to build up to the threshold concentration to trigger CICR in these cells; and (2) the supply of IP3 from these cells to each neighboring ones is reduced.

In our local propagation analysis we assume that the connectivity in a local two–hop neighborhood of astrocyte **X** is acyclic, like in a k_X-ary tree, where k_X is the degree of cell **X** (Figure S4B). We then characterized ICW propagation through cell **X** as the minimum number of astrocytes N_θ in second shell of the k_X-ary tree that has to be activated to also activate cell **X**. Simulations were performed by stimulation of a variable number of astrocytes in the second shell of the k_X-ary tree, with at most one astrocyte stimulated in the periphery of each cell connected with **X** (*green squares*), while the remainder of the cells in the periphery were forced not to activate (*red triangles*, N_s cells per branch) by coupling

them to N_s cells whose intracellular IP_3 concentration was clamped at resting value (i.e. $I = 0.3046\ \mu$M). In agreement with the above analysis, it may be seen from Figure S5A that N_θ depends on the number of unactivated cells (N_s) in the second shell of the tree, almost linearly increasing with them (*dashed lines*). Moreover, the larger the k_X the larger the number of peripheral astrocytes that needed to be stimulated to activate **X**. Larger values of k_X in fact corresponded to more connections between **X** and its neighboring cells, and thus to a larger number of unactivated cells in the network. Therefore, more IP3 had to be supplied to **X** (by stimulation of more cells in the periphery) to compensate for IP3 diffusion from **X** to its unactivated neighbors.

When one cell i, amongst the k_X that are connected to cell **X** gets activated, both its (N_s) unactivated peripheral neighbors and cell **X** act as IP_3 sinks. Thus, cell **X** gets an IP_3 quantity incoming from i that is $Q_i = Q_0/(N_s + 1)$, where Q_0 denotes the IP_3 quantity given by an activated cell (assuming that it doesn't depend on its connectivity). This assumption is supported by measures of outgoing IP_3 during propagation in full networks, for a degree $k > 2$ the quantity of IP_3 given by an activated cell to all its neighbors is nearly constant across all k values (Figure S6). When N_a branches are stimulated among the k_X ones, the total quantity of IP_3 received by cell **X** thus reads:

$$Q_X = \sum_{i=1}^{k_X} Q_i = \frac{N_a Q_0}{N_s + 1} \tag{S10}$$

Accordingly, the normalized threshold IP_3 quantity required to activate **X** (i.e. the minimum normalized IP_3 quantity $\psi_X = Q_X/Q_0$ received by **X**) can be defined as:

$$\psi_\theta = \min_{Q_X} \psi_X = \min_{Q_X} \frac{Q_X}{Q_0} = \frac{N_\theta}{N_s + 1} \tag{S11}$$

Figure S5 shows ψ_θ as a function of the degree of the central cell **X** (k_X), for both nonlinear (Figure S5B) and linear IP_3 diffusion (Figure S5C), respectively for different values of threshold IP3 gradient (I_θ) and GJC permeability (F). Regardless of the diffusion approximation and the value of F or I_θ, the larger k_X the larger ψ_θ. Larger k_X values in fact imply more cells in the network that act as potential IP_3 sinks so that a larger number of stimulated cells is required to allow Ca^{2+} waves to propagate from the periphery to the center.

On the other hand, the threshold stimulus ultimately depends on the IP_3 diffusion rate. In the linear diffusion approximation (Figure S5C), less cells need to be stimulated as GJC permeability increases, since larger F values ease IP_3 diffusion throughout all network and thus facilitate Ca^{2+} wave propagation (compare $F = 0.1$ s^{-1} with $F = 0.25$, i.e. *pink* vs. *lime green curves*). Yet, if F is too large, the ensuing fast IP_3 diffusion prevents IP_3 from accumulating in unactivated cells making it harder to trigger CICR there, unless a larger number of peripheral cells is stimulated. This explains why for $F > 0.25$ s^{-1} in Figure S5C, ψ_θ is larger than for $F < 0.25$ s^{-1}.

Similar arguments also hold true in the nonlinear diffusion approximation (Figure S5B) when the threshold gradient for diffusion is as low as $I_\theta < 0.3\ \mu$M. In these conditions in fact, the ensuing IP_3 flux becomes essentially linear for small IP_3 gradients (equation (S6)). On the other hand, for $I_\theta > 0.3\ \mu$M, ψ_θ is minimal and constant for a large range of k_X values (*pink* and *purple lines*). Due to the high value of I_θ, and differently from the linear scenario, IP_3 can diffuse to and accumulate in one cell i of the k_X ones around **X**, only if the periphery connected to this cell is stimulated. If not, then it is $J_i = 0$ independently of other inputs to other branches of the network, as if the i-th branch was disconnected. Hence, for increasing k_X it makes no difference for the activation of **X** how many cells of the k_X neighbors are unactivated, as far as the minimal number of activated cells that guarantees activation of **X** exists.When I_θ gets as high as 0.5 μM, diffusion starts getting impaired. One must stimulate a larger number of cells to activate **X**, which accounts for values of ψ_θ with $I_\theta = 0.5\ \mu$M that are larger than those for $I_\theta = 0.4\ \mu$M.

The above results are summarized in Figure S5D which considers the slope γ ($\mathrm{d}\psi_\theta/\mathrm{d}k_X$) of the linear

fit of ψ_θ vs. k_X as a function of GJC permeability (F). It may be seen that for $F \geq 0.8$, the slope is steeper in the linear approximation of IP3 diffusion rather than in the nonlinear one, regardless of the value of the IP3 diffusion threshold. This agrees with the observation that for such F values, and the same value of k_X, cell X can only be activated by stimulating a number of peripheral cells that is larger in the linear diffusion approximation with respect to the nonlinear case. In the nonlinear approximation in fact, the existence of a threshold I_θ for intercellular IP3 diffusion prevents rapid flow of IP3 out of cell X due to large values of F, ultimately securing IP3 accumulation and CICR thereby.

S3.2 Whole–network propagation

The results of the above analysis on the mechanisms of regulation of ICW propagation are of general validity and also hold true in larger astrocyte networks. This may be readily seen in the panels of Figure 3 whereby, independently of network topology, the larger the network mean degree $\langle k \rangle$ the lesser the extent of propagation, as quantified by the mean number of cells activated during propagation (N_{act}). In particular, in agreement with results from the local analysis previously shown in Figure S5C and Figure S5D, the extent of propagation was largely reduced even when IP_3 diffusion was linearly approximated by diffusion rates close to $F = 0.25$ s^{-1} (lowest γ slope for linear diffusion but still higher than for nonlinear diffusion with $I_\theta = 0.3$ μM as shown in Figure S5D). On the contrary, in the approximation of nonlinear IP_3 diffusion, values of the threshold for intercellular IP3 diffusion as large as $I_\theta \geq 0.4$ μM generally increased the propagation extent (compare Figure S7B with Figure 3A).

The above analysis of local propagation may be extended to propagation in full networks considering the normalized IP_3 quantity Ψ_i received by an activated astrocyte i during ICW propagation in the full network. This quantity is similar to ψ_X above in the local analysis. We considered cells on the ICW propagation front, so that all their neighbors were either topologically closer to the stimulated cell or never got activated. In analogy with ψ_X (Section S3.1) we computed Ψ_i as:

$$\Psi_i = \max_{t_i^{act}} \psi_i(t_i^{act}) = \max_{t_i^{act}} \sum_{j \in \mathcal{N}_i} \beta_j(t_i^{act}) = \max_{t_i^{act}} \sum_{j \in \mathcal{N}_i} \frac{A_j(t_i^{act} - \Delta t, t_i^{act})}{\sum_{k \in \mathcal{N}_j} (1 - A_k(t_j^{act} - \Delta t, t_j^{act}))} \tag{S12}$$

with t_i^{act} the times at which cell i was activated; $\beta_j(t_i^{act})$ the normalized quantity of IP_3 going out of cell j and to each of its neighbors before the activation of i at t_i^{act}; $A_j(t_1, t_2) = 1$ if cell j was activated between time t_1 and t_2 and 0 otherwise; Δt a time window whose length is of the order of the transmission time between cells during an ICW. Then, we estimated the normalized quantity of IP_3 $\overline{\Psi_i}$ received by a cell i connected to cells on the ICW front but that *never* got activated, as:

$$\overline{\Psi_i} = \sum_{j \in \mathcal{N}_i} \beta_j(t_j^{act}) = \sum_{j \in \mathcal{N}_i} \frac{1}{\sum_{k \in \mathcal{N}_j} (1 - A_k(t_j^{act} - \Delta t, t_j^{act}))} \tag{S13}$$

These measurements (Figure S8A) show that, for astrocytes on the ICW front, spatially constrained networks (Regular in *light green* and link radius in *dark green*) display $\langle \Psi \rangle$ values above the critical value ψ_θ inferred from the local analysis (*black dashed line*, plotted using $k_X = \langle k \rangle$). On the contrary, and as emphasized on Figure S8B, spatially-unconstrained networks (low L values for e.g. Erdős-Rényi networks in *pink* and spatial scale free networks in *dark blue*) are associated with low $\langle \Psi \rangle$ values, comforting that long-distance links impair ICW propagation by decreasing the IP_3 quantity received by cells on the ICW front. Whole network propagation was thus strongly correlated (see Figure S8C) with the distance between the received IP_3 quantity $\langle \Psi \rangle$ and the threshold quantity ψ_θ required for activation; regardless of whether the networks are spatially constrained or not, increasing values of this distance were consistently associated with increasing propagation extent as quantified by the number of activated cells N_{act}. In

agreement with the local analysis, astrocytes that were never activated despite being connected to the ICW front received $\langle\overline{\Psi}\rangle < \psi_\theta$ for all networks (see Figure S8D) except the ones displaying regenerative waves (regular in *light green* and cubic lattice, not shown on the figure because all the cells were activated). Since the correlation between local IP_3 quantities $\langle\Psi\rangle$ and ICW extent N_{act} was obtained whatever the GJC type and parameters (data not shown), we considered that this demonstrates that the main mechanisms from our local analysis still control ICW propagation in full networks.

The effects of shortcuts (or long–distance links) on ICW propagation in the network portion originally considered in Figure S4A, are further detailed in Figure S9. As shown in Figure S9, the replacement of the *grey* link between cell A and another cell on the wave front (marked by '*') by a long–distance connection between cell A and some other cell E in a remote part of the network, hinders activation of further cells such as C and D that lie on the path of ICW propagation. Compared in fact with the original network (Figure S4A), the shortcut between A and E adds a further pathway for IP3 diffusion out of cell A, thus decreasing the amount of IP3 that either cell C or D may receive from it, and ultimately diminishes the likelihood of activation of these latter cells by cell A. Similar arguments also hold if the same shortcut were added in place of the connection between A and C (Figure S9B). In this case, the probability of activation of C is also reduced because less IP3 diffuses to this cell (due to the missing connection with A), so that, ICW propagation is generally hampered. In conclusion, the existence of the long distance connection between cells A and E may equivalently be thought to smear the IP3 supplied by A to a larger portion of the network, preventing its accumulation up to the CICR threshold in individual cells and thus causing early propagation failures.

S3.3 Influence of the shell structure

Given a fraction ρ of activated astrocytes in shell r, the probability for an unactivated astrocyte in r to be connected to none of the ρN^r activated astrocytes is:

$$P_0 = \left(1 - \frac{2W^r}{N^r(N^r-1)}\right)^{\rho N^r} \tag{S14}$$

with $2W^r/(N^r(N^r-1))$ the probability of connection of two distinct nodes in shell r. The probability for an unactivated astrocyte in r to be connected to at least one activated astrocyte is thus, using Taylor expansion at order 1 (as for all the networks in our study $2W^r/(N^r(N^r-1)) \ll 1$):

$$P_1 = 1 - P_0 \approx 1 - \left(1 - \rho N^r \frac{2W^r}{N^r(N^r-1)}\right) = \frac{2\rho W^r}{N^r-1} \tag{S15}$$

The IP_3 quantity Ψ^r_{out} received by nodes of shell $r+1$ derived in the main text confirms, as can be seen on Figure S10A, that the absence of intra-shell links in lattice networks (*purple*) allow them to give higher levels of IP_3 to the nodes in the next shell, ensuring regenerative propagation. For the same degree distribution $k = 6$, regular networks send much less IP_3 to the next shell, resulting in early propagation failure. Interestingly, these Ψ^r_{out} values are ordered on Figure S10A as on Figure 3: high propagation extent N_{act} is associated to high Ψ^r_{out} values, hinting that shell structure may drive wave propagation.

To test this hypothesis, we devised a simplified propagation model that only takes into account the shell structures of networks, discarding all other specific topological characteristics. Average shell structures were obtained by taking the central node of each network as the reference node of the shell decomposition. The values N^r, E^r and W^r were taken, for each shell radius r, as averages across 20 networks of the same type. We then devised a simplified propagation model by using the IP_3 quantity Ψ^r_{out} given by astrocytes of shell r to neighbouring nodes and rescaling it by the fraction of links going to the next shell $r+1$ among all the links going to unactivated nodes. We thus defined the corrected IP_3 quantity $\hat{\Psi}^r_{out}$ received by nodes of shell $r+1$ by:

$$\hat{\Psi}^r_{out} = \frac{E^r}{E^{r-1} + E^r + 2\rho^r(1-\rho^r)W^r} \times \Psi^r_{out} \tag{S16}$$

With ρ^r being the fraction of activated astrocytes in shell r. As shown in Section S3.1, the IP_3 quantity needed to activate a node follows $\psi_\theta(k) = ck + d$ with k the degree of the node. As we only want to take shell structures into account (and not the detailed degree distributions), we used the mean degree $\langle k \rangle$ of networks to compute ψ_θ. Knowing the fraction of activated astrocytes in shell r, we approximated its value for shell $r+1$ as being a simple sigmoidal function of the distance between the quantity of IP_3 received by nodes of this shell and the quantity ψ_θ needed to activate a node:

$$\rho^{r+1} = \frac{1}{2}\left(\tanh\left(\frac{\hat{\Psi}^r_{out} - \psi_\theta(\langle k \rangle)}{\delta}\right) + 1\right) \quad \text{(S17)}$$

With δ a parameter controlling the steepness of the transition between sub and supra-threshold IP_3 quantities. ρ^r can then be computed recursively for each shell r by imposing the activation of the first shell $\rho^0 = \rho^1 = 1$ which is observed in the full ChI model simulations. Propagation was considered finished at shell R if $\rho^R N^R < 1$ and the total number of activated astrocytes was thus computed with:

$$N_{sim} = \sum_{r=0}^{R} \rho^r N^r \quad \text{(S18)}$$

Parameters c, d and δ were identical for all network types and were estimated using the CMA-ES optimization procedure [40] (taking the distance between N_{sim} and N_{act} as the function to minimize). Values are reported in Table S2.

We computed N_{sim} for all network types presented in Figure 1 and compared it on Figure S10B to actual propagation extent N_{act}. N_{sim} was close to N_{act} for all network types, showing that propagation extent can be fully accounted for by the shell structure of networks, without taking into account additional topological characteristics.

References

1. Malarkey E, Ni Y, Parpura V (2008) Ca^{2+} entry through TRPC1 channels contributes to intracellular Ca^{2+} dynamics and consequent glutamate release from rat astrocytes. Glia 56: 821–835.

2. Shigetomi E, Tong X, Kwan K, Corey D, Khakh B (2011) TRPA1 channels regulate astrocyte resting calcium levels and inhibitory synapse efficacy via GAT-3. Nat Neurosci 15: 70–80.

3. Verkhratsky A, Rodríguez JJ, Parpura V (2012) Calcium signalling in astroglia. Mol Cell Endocrinol 353: 45–56.

4. Volterra A, Meldolesi J (2005) Astrocytes, from brain glue to communication elements: the revolution continues. Nat Rev Neurosci 6: 626–640.

5. Nimmerjahn A (2009) Astrocytes going live: advances and challenges. J Physiol 587: 1639-1647.

6. Ramos-Franco J, Bare D, Caenepeel S, Nani A, Fill M, et al. (2000) Single-channel function of recombinant type 2 inositol 1,4,5-trisphosphate receptor. Biophys J 79: 1388–1399.

7. Iino M (1990) Biphasic Ca^{2+}-dependence of inositol 1,4,5-trisphosphate-induced Ca^{2+} release in smooth muscle cells of the guinea pig *Taenia caeci.* J Gen Physiol 95: 1103-1112.

8. Bezprozvanny I, Watras J, Ehrlich BE (1991) Bell-shaped calcium-response curves of Ins(1,4,5)P_3- and calcium-gated channels from endoplasmic reticulum of cerebellum. Nature 351: 751-754.

9. Watras J, Bezprozvanny I, Ehrlich BE (1991) Inositol 1,4,5-trisphosphate-gated channels in cerebellum: presence of multiple conductance states. J Neurosci 11: 3239-3245.

10. Kaftan EJ, Erlich BE, Watras J (1997) Inositol 1,4,5-trisphosphate ($InsP_3$) and calcium interact to increase the dynamic range of $InsP_3$ receptor-dependent calcium signaling. J Gen Physiol 110: 529-538.

11. Mak D, McBride S, Foskett J (2003) Spontaneous channel activity of the inositol 1,4,5-trisphosphate ($InsP_3$) receptor ($InsP_3R$). Application of allosteric modeling to calcium and $InsP_3$ regulation of $InsP_3R$ single-channel gating. J Gen Physiol 122: 583–603.

12. Berridge MJ, Bootman MD, Roderick HL (2003) Calcium signalling: dynamics, homeostasis and remodelling. Nat Rev Mol Cell Biol 4: 517-529.

13. De Pittà M, Volman V, Berry H, Parpura V, Liaudet N, et al. (2012) Computational quest for understanding the role of astrocyte signaling in synaptic transmission and plasticity. Front Comp Neurosci 6: 98.

14. Zhang BX, Zhao H, Muallem S (1993) Calcium dependent kinase and phosphatase control inositol-1,4,5-trisphopshate-mediated calcium release: modification by agonist stimulation. J Biol Chem 268: 10997-11001.

15. Sims CE, Allbritton NL (1998) Metabolism of inositol 1,4,5-trisphosphate and inositol 1,3,4,5-tetrakisphosphate by the oocytes of *Xenopus laevis*. J Biol Chem 273: 4052-4058.

16. Rebecchi MJ, Pentyala SN (2000) Structure, function, and control of phosphoinositide-specific phospholipase C. Physiol Rev 80: 1291-1335.

17. Pittà MD, Goldberg M, Volman V, Berry H, Ben-Jacob E (2009) Glutamate regulation of calcium and IP3 oscillating and pulsating dynamics in astrocytes. J Biol Phys 35: 383–411.

18. Scemes E, Giaume C (2006) Astrocyte calcium waves: What they are and what they do. Glia 54: 716–725.

19. Guthrie PB, Knappenberger J, Segal M, Bennett CAC M V L, Kater SB (1999) ATP released from astrocytes mediates glial calcium waves. J Neurosci 19: 520-528.

20. Haas B, Schipke CG, Peters O, Shl G, Willecke K, et al. (2006) Activity-dependent ATP-waves in the mouse neocortex are independent from astrocytic calcium waves. Cereb Cortex 16: 237-246.

21. Giaume C, Koulakoff A, Roux L, Holcman D, Rouach N (2010) Astroglial networks: a step further in neuroglial and gliovascular interactions. Nat Rev Neurosci 11: 87–99.

22. Carmignoto G (2000) Reciprocal communication systems between astrocytes and neurones. Prog Neurobiol 62: 561-581.

23. Kettenmann H, Ransom BR (2004) Neuroglia, The concept of neuroglia: A historical perspective. New York: Oxford University Press, 1st edition.

24. Crank J (1980) The mathematics of diffusion. USA: Oxford University Press, 2 edition.

25. Sneyd J, Charles AC, Sanderson MJ (1994) A model for the propagation of intracellular calcium waves. Am J Physiol 266: C293-C302.

26. Falcke M (2004) Reading the patterns in living cells: the physics of Ca^{2+} signaling. Adv Phys 53: 255-440.

27. Witcher M, Kirov S, Harris K (2007) Plasticity of perisynaptic astroglia during synaptogenesis in the mature rat hippocampus. Glia 55: 13-23.

28. Pivneva T, Haas B, Reyes-Haro D, Laube G, Veh R, et al. (2008) Store-operated Ca^{2+} entry in astrocytes: different spatial arrangement of endoplasmic reticulum explains functional diversity in vitro and in situ. Cell Calcium 43: 591–601.

29. Nagy JI, Rash JE (2000) Connexins and gap junctions of astrocytes and oligodendrocytes in the CNS. Brain Res Rev 32: 29–44.

30. Harris AL (2001) Emerging issues in connexin channels: biophysics fills the gap. Quarterly Rev Biophys 34: 325-472.

31. Kwak B, Hermans MMP, DeJonge H, Lohmann S, Jongsma HJ, et al. (1995) Differential regulation of distinct types of gap junction channels by similar phosphorylating conditions. Mol Biol Cell 6: 1707-1719.

32. Lampe PD, Tenbroek EM, Burt JM, Kurata WE, Johnson RG, et al. (2000) Phosphorylation of connexin43 on serine368 by protein kinase C regulates gap junctional communication. J Cell Biol 149: 1503-1512.

33. Goldberg M, De Pittà M, Volman V, Berry H, Ben-Jacob E (2010) Nonlinear gap junctions enable long-distance propagation of pulsating calcium waves in astrocyte networks. PLoS Comput Biol 6: e1000909.

34. Jourdain P, Bergersen LH, Bhaukaurally K, Bezzi P, Santello M, et al. (2007) Glutamate exocytosis from astrocytes controls synaptic strength. Nat Neurosci 10: 331–339.

35. Panatier A, Vallée J, Haber M, Murai K, Lacaille J, et al. (2011) Astrocytes are endogenous regulators of basal transmission at central synapses. Cell 146: 785-798.

36. Fiacco TA, McCarthy KD (2004) Intracellular astrocyte calcium waves in situ increase the frequency of spontaneous AMPA receptor currents in CA1 pyramidal neurons. J Neurosci 24: 722–732.

37. Boccaletti S, Latora V, Moreno Y, Chavez M, Hwang DU (2006) Complex networks: structure and dynamics. Phys Rep 424: 175 - 308.

38. Costa LdF, da Rocha LEC (2006) A generalized approach to complex networks. Eur Phys J B 50: 237-242.

39. Feldt S, Wang JX, Shtrahman E, Dzakpasu R, Olariu E, et al. (2010) Functional clustering in hippocampal cultures: relating network structure and dynamics. Phys Biol 7: 046004.

40. Hansen N (2006) The cma evolution strategy: A comparing review. In: Lozano J, Larraaga P, Inza I, Bengoetxea E, editors, Towards a New Evolutionary Computation, Springer Berlin Heidelberg, volume 192 of *Studies in Fuzziness and Soft Computing*. pp. 75-102.

1 Supplementary Tables

Table S1. UAR propagation model parameters.

Symbol	Description	Values	Units
τ_U	Average time needed to activate an astrocyte	7	s
τ_A	Average activation time of an astrocyte	9	s
τ_R	Average refractory time of an astrocyte	6.5	s
a	Slope of the relationship between k_i and ϑ_i	0.02	-
b	Intercept of the relationship between k_i and ϑ_i	0.205	-

Table S2. Supplementary material parameters.

Symbol	Description	Values			Units
		min	step	max	
	ICW Visualization				
α	Distance rescaling factor		10		μm^{-}1
β	Default length of intercellular space		35		μm
γ	Intensity of darkening of extracellular space		4		-
	Local ICW Propagation				
k_X	Degree of cell X (center cell)	3	1	15	-
N_a	Number of stimulated branches	3	1	15	-
N_s	Number of sink neighbors in each branch	0	1	5	-
	Shell analysis				
c	Slope of the relationship between k and ψ_θ		0.0083		-
d	Intercept of the relationship between k and ψ_θ		0.1519		-
δ	Steepness of transition between sub and supra-threshold IP_3		0.0561		-

Supplementary figures

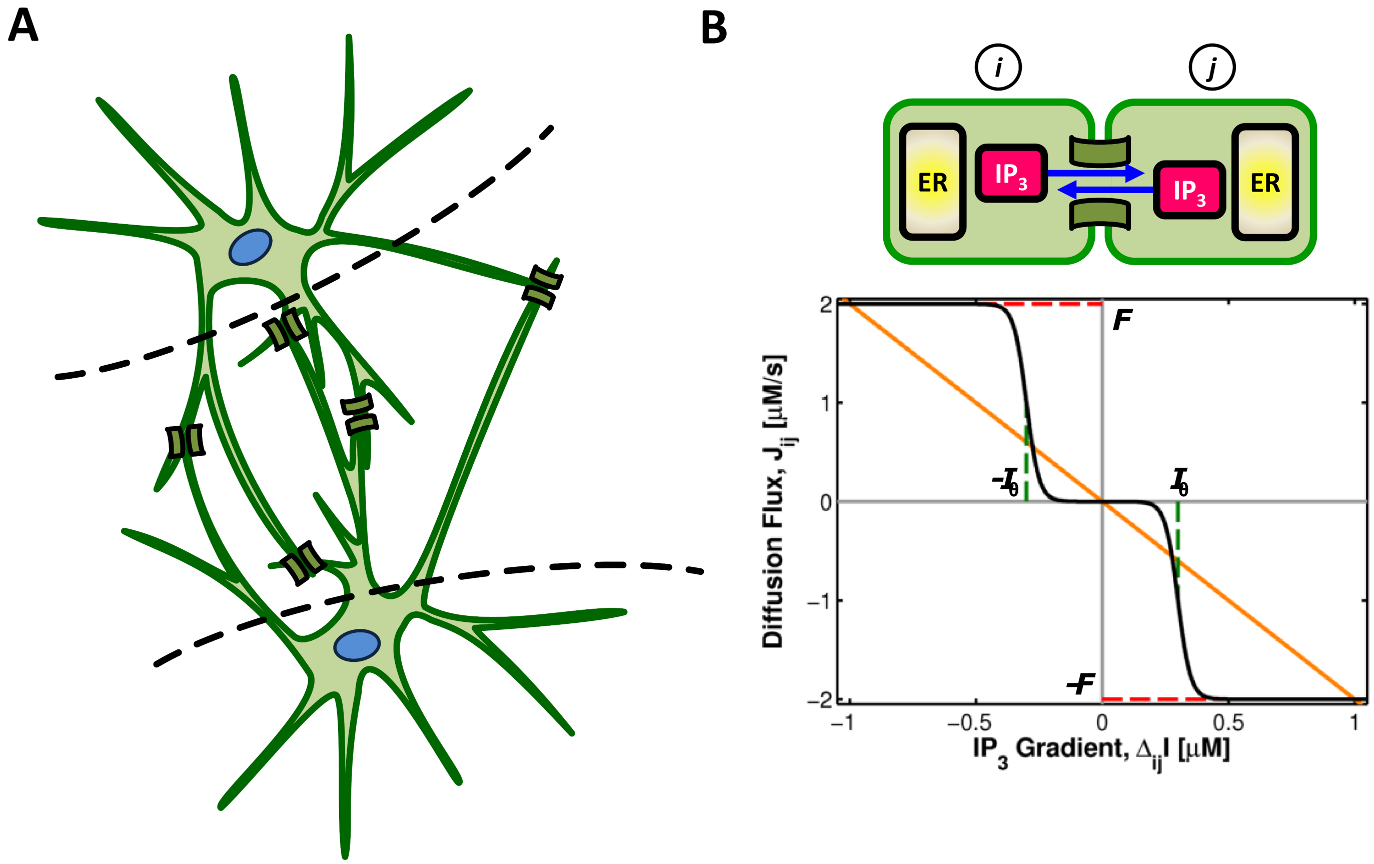

Figure S1. Intercellular IP_3 diffusion. **A** Intercellular Ca^{2+} wave propagation in astrocyte networks relies on IP_3 diffusion across cell somata. The diffusion pathway however is complex and depends on the intricate morphology of astrocyte processes and the existence of discrete sites of intercellular communication through GJCs. These factors could create a diffusion barrier between cell somata (*dashed black lines*) which would ultimately result in nonlinear diffusion of IP_3 among cells. **B** Comparison between linear (*orange line*) vs. nonlinear diffusion fluxes (*black line*), for GJC–mediated IP_3 exchange between astrocytes. In the nonlinear approximation a threshold gradient IP_3 concentration between cells (I_θ, *dashed green lines*) must exist for IP_3 to effectively diffuse from one cell to another. Maximal GJC permeability sets the slope of the flux in the linear description while it dictates the maximum flux in the nonlinear approximation (*dashed red lines*). Parameters as in Table 1.

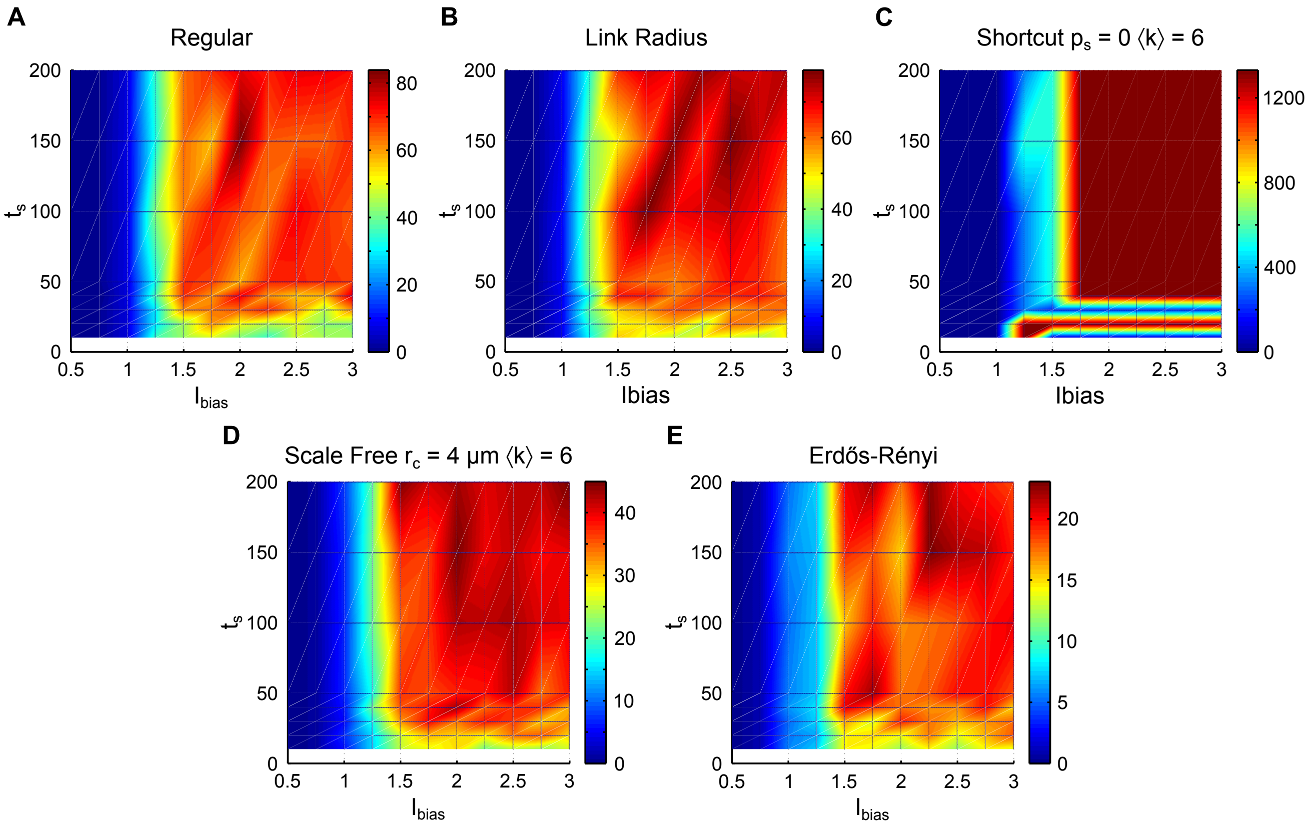

Figure S2. Protocol of astrocyte stimulation used in the simulations. In all astrocyte networks considered in this study, ICW propagation was triggered by a step IP_3 concentration gradient (I_{bias}) applied to the astrocyte at the center of the network. The duration (t_s) of the step was fixed at 200 s to ensure that ICW could unolfd to their full extent. This extent was determined by looking at the number of astrocytes activated by an ICW (N_{act}, *colorbars*) as a function of stimulation time and IP_3 bias. All networks shown in the figure were characterized by $\langle k \rangle = 6$. (**A**) Regular networks with $k = 6$; (**B**) Link Radius networks with $d = 85$ μm ; (**C**) Shortcut networks with $m = 1$ and $p_s = 0$; (**D**) Spatial Scale Free networks with $m_{sf} = 3$ and $r = 4$ μm; and (**E**) Erdős-Rényi with $p = 6/(N-1)$. I_{bias} was varied from 0.5 μM and 3 μM by steps of 0.5 μM; t_s was varied from 10 to 50s by steps of 10s and from 50 to 200s by steps of 50s. The data show the means value of N_{act} for 20 realizations of the same network topology. Note that colorbars vary between subfigures.

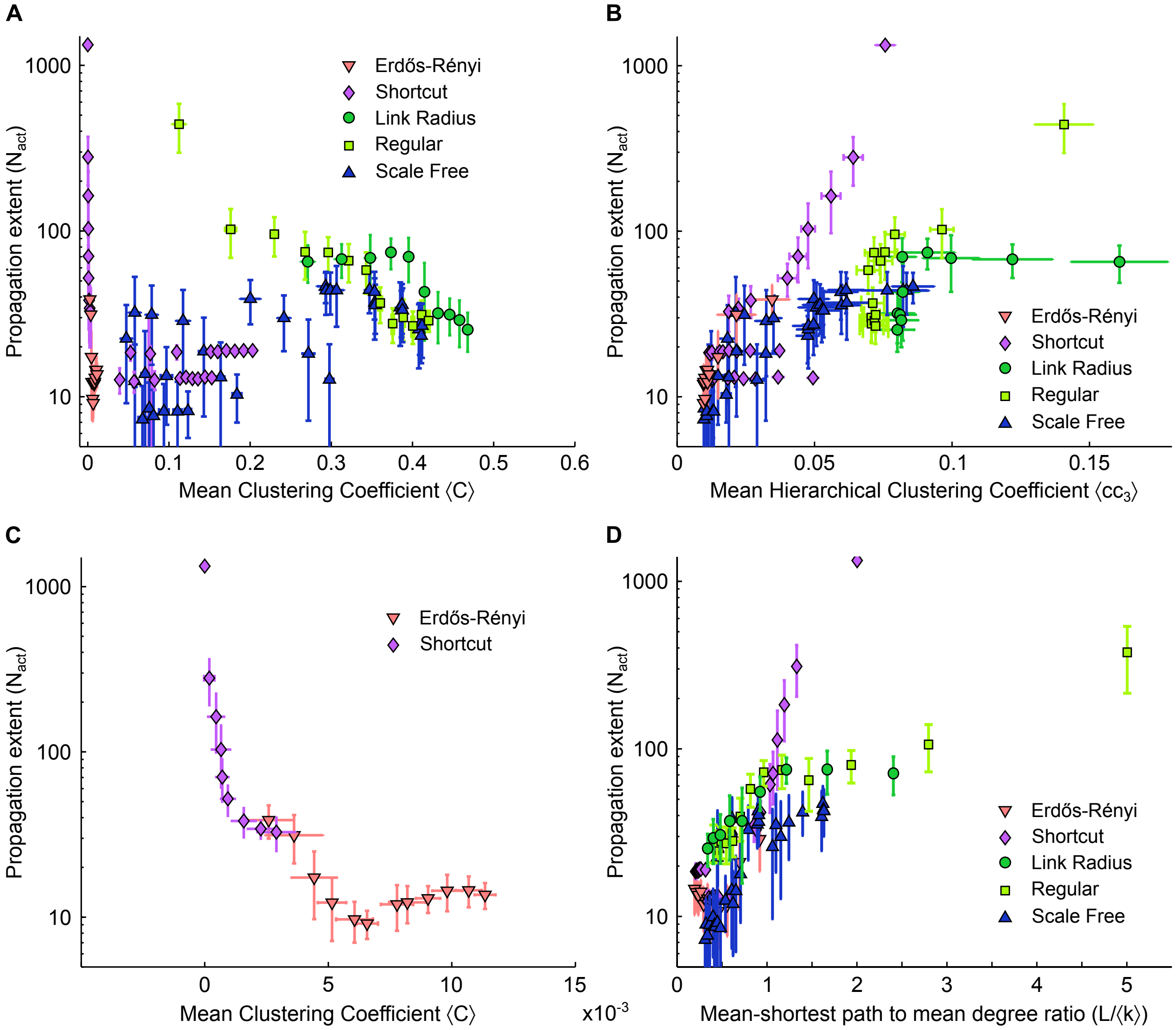

Figure S3. Effect of clustering on ICW propagation. Extent of ICW propagation quantified by the number of activated cells (N_{act}) as a function of the **A** mean clustering coefficient $\langle C\rangle$ and **B** the mean hierarchical clustering coefficient $\langle cc_d\rangle$ with $d = 3$. **C** Zoom on the low $\langle C\rangle$ values (appearing to form a vertical line in **A**). Differently from other network parameters, such as mean degree and mean shortest path, the effect of cell clustering on the ICW propagation is complex and does not reveal any specific trend. **D** The mean-shortest path to mean degree ratio $L/\langle k\rangle$ does not explain the propagation extent better than $\langle k\rangle$ and L taken separately. Data are plotted as mean±std for 20 different network realizations with the same topology. Clustering coefficients were computed according to Refs [37–39].

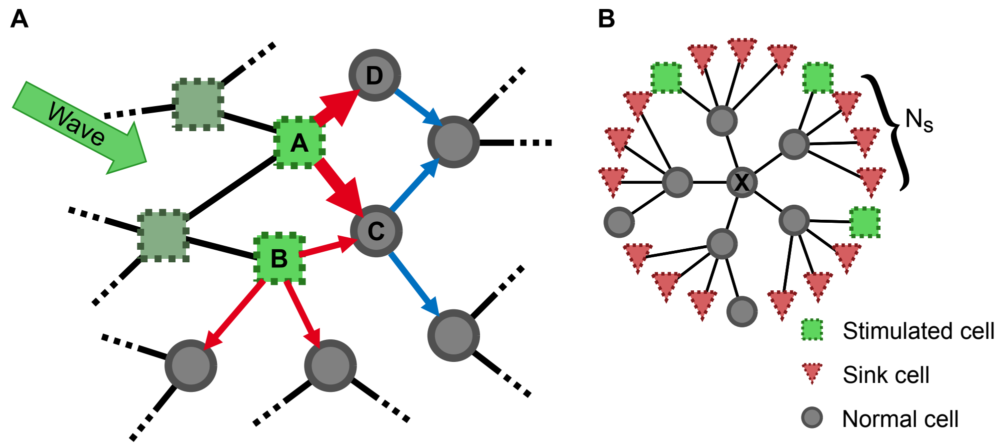

Figure S4. Local propagation in astrocyte networks. **A** Ca^{2+} propagation to one astrocyte depends on the number of its activated connected neighbors. Unactivated cells act in fact as IP_3 sinks, thus hindering IP_3 accumulation and CICR regeneration. In this fashion, an activated cell like B that is connected to more unactivated cells than A, is less likely to allow propagation to its neighbors than the latter. For the same reason, an unactivated cell like D, which is connected to a smaller number of unactivated cells than C, is more likely to get activated. **B** Locally, ICW propagation through astrocyte networks may be approximated considering Ca^{2+} propagation from the periphery to the central cell X in a two–hop k_X-ary tree. Accordingly, activation of cell X can be studied varying (1) the number N_a of stimulated branches out of k_X branches of the tree (*green squares*, a maximum of one per branch) and (2) the number N_s of sink cells (*red triangles*) in each branch. Astrocyte parameters as in Table 1; sink cells are coupled to N_s virtual cells (not represented on the figure) with $I_{bias} = 0.3046$ μM (equilibrium value). Small network parameters varied as in Table S2.

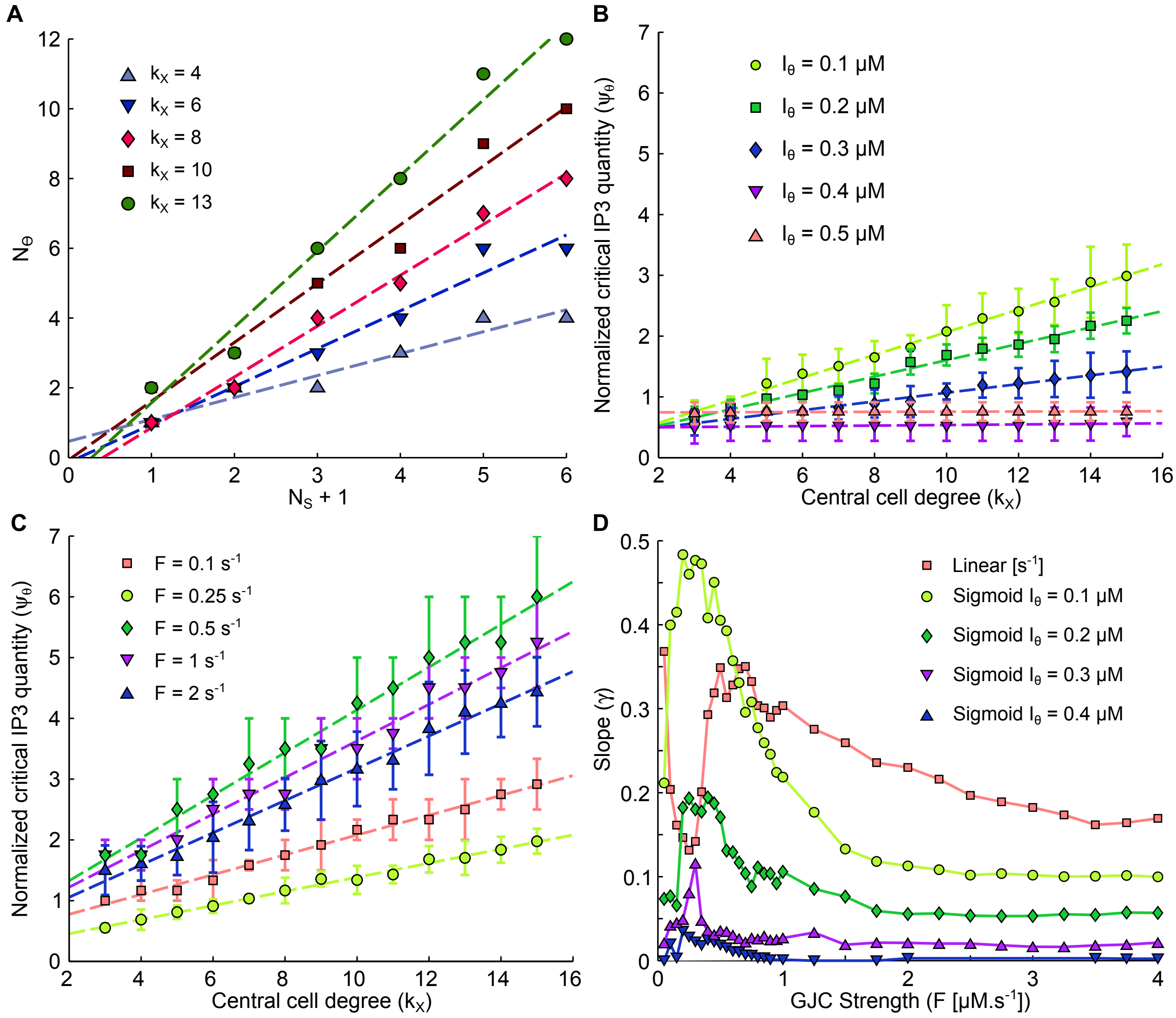

Figure S5. Threshold stimuli for propagation depend on local connectivity. **A** The minimum number of branches (N_θ) in the k_X-ary tree (Figure S4B) that have to be activated to allow ICW propagation through cell X, increases with the number of unactivated cells (N_s) in an essentially linear fashion (*dashed lines*). For a fixed degree k_X, the slope of this relationship is the threshold normalized IP_3 quantity ψ_θ needed to activate cell X; it generally increases in a linear fashion (*dashed lines*) with the degree k_X of the central cell. This holds true for both **B** nonlinear and **C** linear intercellular IP_3 diffusion, although the linear dependence on k_X is different in the two cases. **D** Except for very low threshold values for IP_3 intercellular diffusion, the slope γ ($\mathrm{d}\psi_\theta/\mathrm{d}k_X$) of the linear fit of ψ_θ vs. k_X is generally steeper for linear than nonlinear IP_3 diffusion. That is, for the same central cell degree k_X, the number of peripheral cells that need to be activated to ensure ICW propagation through cell X is larger with linear IP_3 diffusion than with nonlinear one. Note that F is not expressed in the same units for linear (s^{-1}) and nonlinear (μM.s^{-1}) IP_3 diffusion. **B**, **C**: Data points±errorbars correspond mean±std values of ψ_θ computed for $N_s = \{0, \ldots, 5\}$. Stimulus protocol and model parameters as in Figure S4.

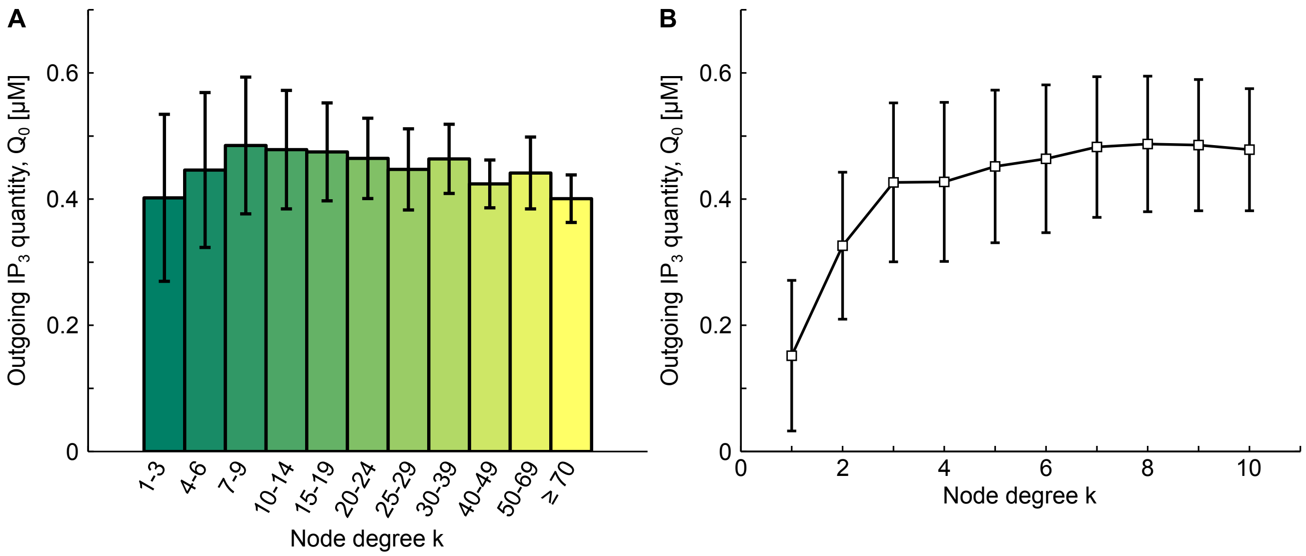

Figure S6. Outgoing IP_3 quantity Q_0 doesn't depend on node connectivity. Outgoing IP_3 quantities Q_0 were obtained by integrating outgoing IP3 fluxes J_{ij} across all neighbors of each activated cell during 7s (propagation time as determined in Figure 5). **A** Q_0 is almost constant for all node degrees k. **B** Detailed values for each degree k; for $k \leq 2$, Q_0 is however lower as IP_3 can only leak to 1 or 2 cells. Stimulated nodes or neighbors of stimulated nodes were not taken into account as their outflux is biased by stimulation. Data±errorbars: mean±std on all activated nodes of degree k for 20 realizations of networks of same topology and networks parameters as in Table 2. Model parameters as in Table 1.

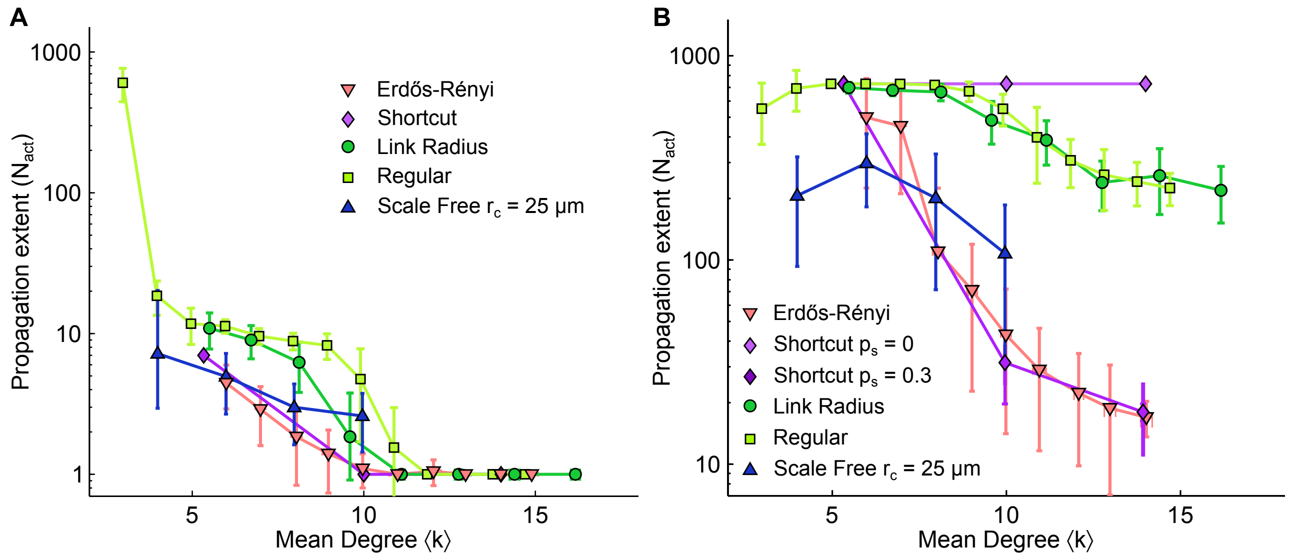

Figure S7. Dependence of ICW propagation on IP3 diffusion. **A** Extent of ICW propagation (N_{act}) in astrocyte networks generally decreases with the cell mean degree ($\langle k \rangle$) but it is generally limited to few cells (<10) in the assumption of linear intercellular IP3 diffusion, independently of network topology. The only possible exception is for spatially–constrained networks such as regular networks with $\langle k \rangle \leq 3$. **B** In contrast, the propagation extent is much larger in presence of a threshold (I_θ) for intercellular IP3 diffusion (I_θ). Comparison with Figure 3A in particular, suggests that in the same network, the larger this threshold, the larger N_{act}. Data±errorbars: mean±std on 20 realizations of networks of same topology. **A**: $F = 0.25\ \mathrm{s}^{-1}$; **B**: $I_\theta = 0.5$, $F = 2\ \mu\mathrm{M\,s}^{-1}$. Other parameters as in Figure 3A.

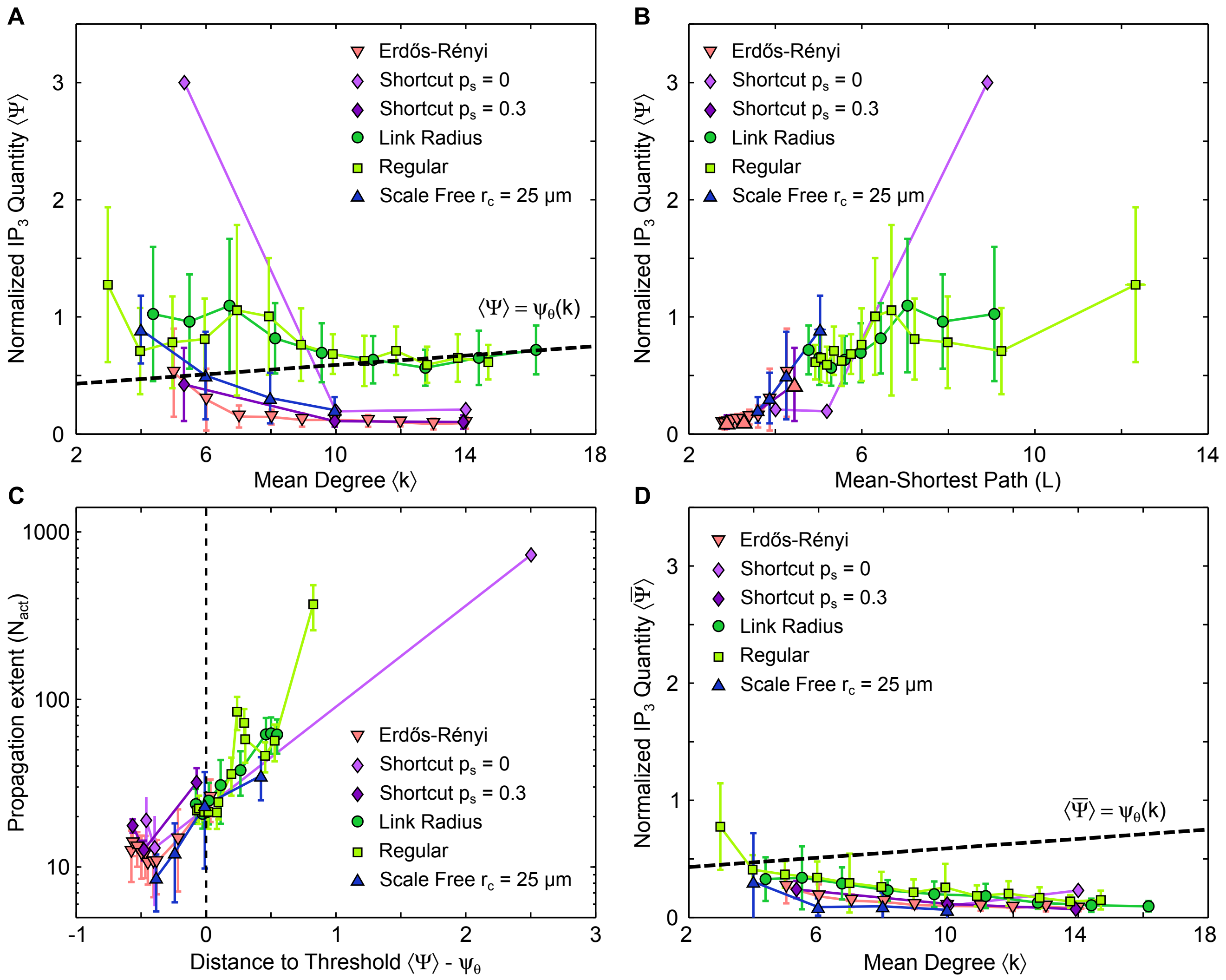

Figure S8. Application of local analysis to whole network propagation. Normalized IP_3 quantity received by cells on the ICW front were plotted against **A** the mean degree $\langle k \rangle$ and **B** the mean-shortest path L for all the networks used in the main analysis. Spatially constrained networks are nearly always above the threshold quantity ψ_θ needed to propagate ICW (reported as the *dashed black line* obtained by linear interpolation of data in *blue* in Figure S5B). Low mean-shortest path is associated to low IP_3 quantity received, accounting for the low propagation extent shown in Figure 3. Accordingly, as depicted on **C**, propagation extent N_{act} is strongly correlated to the difference between received IP_3 quantity $\langle \Psi \rangle$ and threshold IP_3 quantity ψ_θ, proving that the local analysis is still valid when applied to network wide propagation. Cells that were not activated despite being connected to cells on the ICW front received sub-threshold IP_3 quantities $\langle \overline{\Psi} \rangle$ as depicted on **D**. Data points are shown as mean±std for 20 different network realizations of each topology. Parameters as in Table 1.

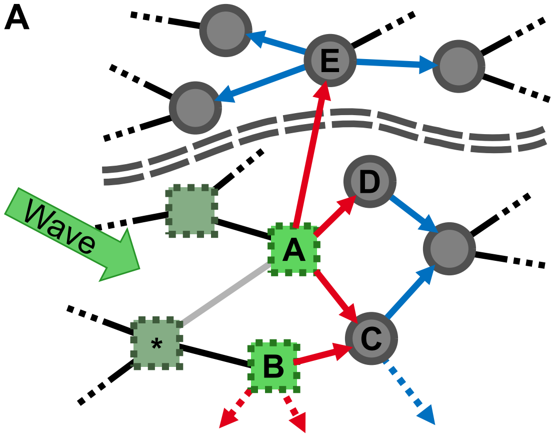

Figure S9. Effect of shortcuts on ICW propagation. Connections (in *grey*) of astrocyte A in the local network of Figure S4A were rewired from cells marked by '*' to a cell E located in a far unactivated area of the same network (separated by *dashed lines*). **A** The additional connection between cells A and E reduces the IP3 supply from cell A to cells C and D hindering ICW propagation to them. **B** Similarly, cell C is supplied by less IP3 if it were not connected with A but only with B due to the existence of the long distance connection between A and E. Long distance connections thus generally hamper ICW propagation, as they reduce IP3 supply to individual cells, counteracting its accumulation up to the CICR threshold.

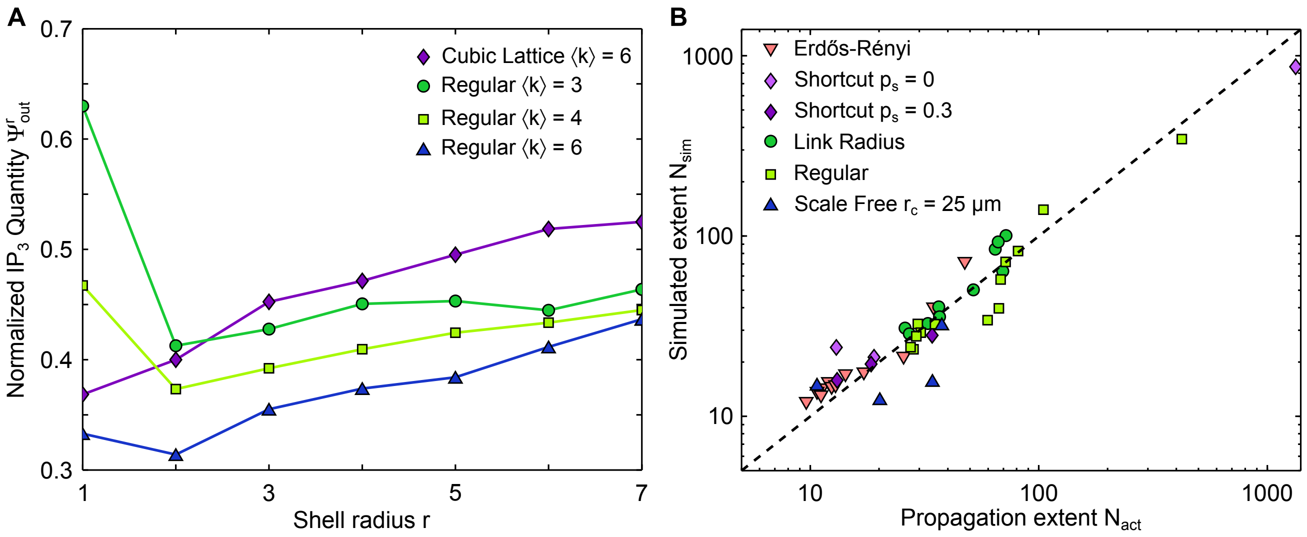

Figure S10. Shell analysis. Shell analysis reveals that differences in ICW propagation may be attributable to connections between cells within the same r-th shell (W^r) in regular networks, which reduce the quantity of IP_3 going to the next shell $r+1$, thus resulting in earlier propagation failures than in cubic lattices. **A** the quantity of IP_3 Ψ^r_{out} received by nodes of shell $r+1$ is much higher, for $r \geq 2$, in cubic lattices (*purple*) than in regular networks (*light green*, *dark green* and *blue*). These values match with the propagation extent, the higher Ψ^r_{out}, the higher N_{act} (see Figure 3). Ψ^r_{out} was computed with $\rho^r = 0.5$ for all shells and was normalized by using $Q_0 = 1$. **B** Propagation can be simulated using only shell decomposition, the resulting simulated extent N_{sim} matches closely with the actual propagation extent N_{act} computed in the full ChI model (correlation coefficient computed in log-log scale is 0.96), emphasizing the crucial role of shell structure in ICW propagation. The dashed line represents the $N_{sim} = N_{act}$ line. Networks parameters as in Table 2. Propagation parameters as in Table S2.

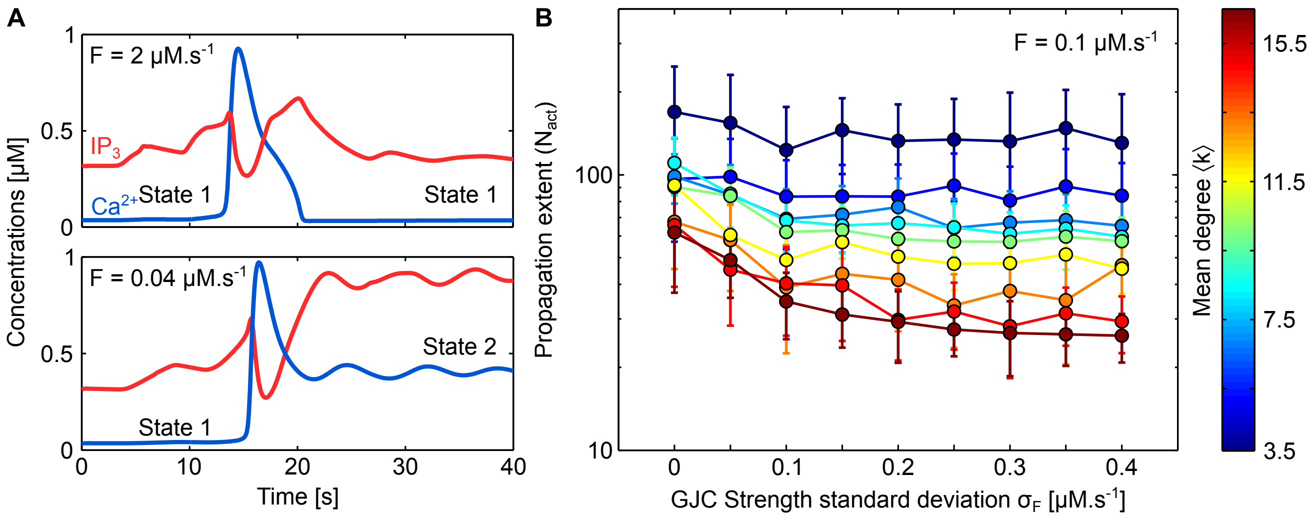

Figure S11. Variations of GJC strength. **A** For low values of GJC strength F, the equilibrium state of astrocytes in networks is switched by the arrival of the ICW from steady-state 1 (low Ca^{2+} values) to steady-state 2 (larger Ca^{2+} values) where the resting concentrations of IP_3 (in *red*) and Ca^{2+} (in *blue*) are much higher. **B** Increasing GJC variability (σ_F) hardly has an effect on low degree networks (*blue* curves) while it decreases ICW extent by up to 50% for high degree networks (*red* curves). All points represent 20 realizations of a given parameter combination. Data points in **B** are color-coded according to the mean degree $\langle k \rangle$ of the networks. Astrocyte and network parameters as in Figure 8.
